# Recent population differentiation in the habitat specialist Glossy Antshrike (Aves: Thamnophilidae) across Amazonian seasonally flooded forests

**DOI:** 10.1002/ece3.7951

**Published:** 2021-07-28

**Authors:** Sofia Marques Silva, Camila C. Ribas, Alexandre Aleixo

**Affiliations:** ^1^ Research Centre in Biodiversity and Genetic Resources CIBIO/InBIO Vairão Portugal; ^2^ Department of Zoology Museu Paraense Emílio Goeldi Belém Brazil; ^3^ Instituto Nacional de Pesquisas da Amazônia INPA Manaus Brazil; ^4^ Finnish Museum of Natural History University of Helsinki Helsinki Finland

**Keywords:** *Igapó*, Phylogeography, population genomics, *Sakesphorus luctuosus*, taxonomy, ultraconserved elements, *Várzea*

## Abstract

We assessed population structure and the spatio‐temporal pattern of diversification in the Glossy Antshrike *Sakesphorus luctuosus* (Aves, Thamnophilidae) to understand the processes shaping the evolutionary history of Amazonian floodplains and address unresolved taxonomic controversies surrounding its species limits. By targeting ultraconserved elements (UCEs) from 32 specimens of *S*. *luctuosus*, we identified independent lineages and estimated their differentiation, divergence times, and migration rates. We also estimated current and past demographic histories for each recovered lineage. We found evidence confirming that *S*. *luctuosus* consists of a single species, comprising at least four populations, with some highly admixed individuals and overall similar levels of migration between populations. We confirmed the differentiation of the Araguaia River basin population (*S. l*. *araguayae*) and gathered circumstantial evidence indicating that the taxon *S*. *hagmanni* may represent a highly introgressed population between three distinct phylogroups of *S*. *luctuosus*. Divergences between populations occurred during the last 1.2 mya. Signs of population expansions were detected for populations attributed to subspecies *S. l*. *luctuosus*, but not for the *S. l*. *araguayae* population. Our results support that *S*. *luctuosus* has had a complex population history, resulting from a high dependence on southeastern “clear water” seasonally flooded habitats and their availability through time. Spatial and demographic expansions toward the western “white water” flooded forests might be related to recent changes in connectivity and availability of these habitats. Our study reinforces the view that isolation due to absence of suitable habitat has been an important driver of population differentiation within Amazonian flooded forests, but also that differences between *várzeas* (“white water” floodplains, mostly in southwestern Amazonia) and *igapós* (“clear water” floodplains, especially located in the east) should be further explored as drivers of micro‐evolution for terrestrial species.

## Graphical abstract

We assessed population structure and the spatio‐temporal pattern of diversification in the Glossy Antshrike *Sakesphorus luctuosus* (Aves, Thamnophilidae) to understand the processes shaping the evolutionary history of Amazonian floodplains and address unresolved taxonomic controversies surrounding its species limits. We found evidence confirming that *S. luctuosus* consists of a single species, comprising at least four populations, with some highly admixed individuals and similar levels of migration between populations. Our study reinforces the view that isolation due to absence of suitable habitat has been an important driver of population differentiation within Amazonian flooded forests, but also that differences between várzeas (“white water” floodplains, mostly in southwestern Amazonia) and igapós (“clear water” floodplains, especially located in the east) should be further explored as powerful drivers of micro‐evolution.

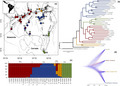

## 1 INTRODUCTION

**FIGURE 1:**
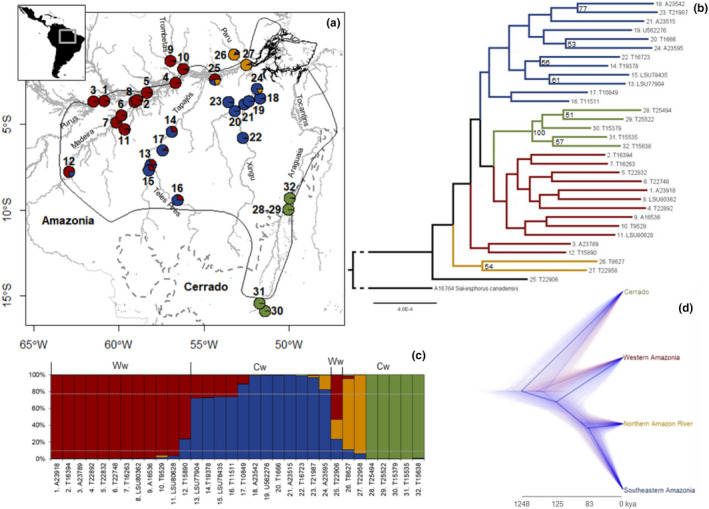
(a) Glossy Antshrike *Sakesphorus luctuosus* known distribution (delimited by the solid black line; BirdLife International, 2016). Amazonia and *Cerrado* are bordered by the dotted gray line and main Amazonian rivers (dark gray lines) are also represented. Only rivers mentioned throughout the text are named. (a–d) Sampled localities are numbered and specimens represented by colors according to their putative population assignment: western Amazonia—red; southeastern Amazonia—blue; north of the Amazon River—orange; and *Cerrado*—green. Specimen T22906 (locality 25, ca. 18 km from Taperinha, *S. l*. *hagmanni* type locality) represents an admixed individual that could not be attributed to either a genetic or geographic population and putatively belongs to *S. l*. *hagmanni*. Individuals attributed to *S. l*. *araguayae* were sampled from localities 28–32. (b) Maximum likelihood tree for *Sakesphorus luctuosus*; only bootstrap values superior to 50 are depicted. (c) STRUCTURE assignment plot for each specimen sequenced considering four clusters. Each vertical bar represents a specimen (see Table S1 for detailed locality information). Correspondence with the type of water for the main rivers of occurrence of each population follows Junk et al. (2011): Ww for white water rivers and Cw for clear water rivers. (d) Species tree excluding highly admixed individuals between distinct population groups. Divergence time estimates for each split are depicted in thousands of years (kya), but not to scale (Fig. S4 for more details on these estimates)

Most swampy and flooded forests are found in the tropics, particularly in the Congo River Basin and northern/northeastern South America, with the most widespread area of inundated forests occurring along rivers within the Amazon River Basin (Lehner & Döll, 2004). Amazonian wetlands cover approximately 14% of the basin (Figure S1a; Albert et al., 2018; Hess et al., 2015) and can be divided and categorized by differences in the physical and chemical composition of the water, soil quality, vegetation type, and degree of flooding (Junk et al., 2011). The Amazon River and its western tributaries, for example, Madeira, Japurá, and Purus rivers (see Figure 1a for geographic reference), have high‐fertility “white waters,” due to the high concentration of Andean sediments, with their associated flooded vegetation called *várzeas*. Eastern rivers, such as the Tapajós and Xingu, are “clear water” rivers, with intermediate fertility. Conversely, the Negro River sub‐Basin and a few other tributaries are constituted by low‐fertility “black waters” (Junk et al., 2011). Both clear and black water rivers drain sediment‐poor terrains from the Brazilian and Guiana shields, and their associated vegetation is named *igapó*.

*Várzeas* and *igapós* occur apparently continuously along the Amazonian rivers and depend on the seasonality of the inundation regimes (Aleixo, 2006; Junk et al., 2011). Limited differentiation within bird species restricted to flooded forests reinforced this view (Aleixo, 2002, 2006; Ribas et al., 2009). However, an analysis of avian endemism along the Solimões‐Amazonas River supported the Negro, Madeira, and Tapajós river mouths as important barriers for many, but not all, flooded forest bird species (Cohn‐Haft et al., 2007). Currently, habitat specialization within flooded forests is recognized to occur and to influence taxa distribution and diversity, albeit with some caveats. Habitats associated with distinct water types seem to hold dissimilar bird communities, but few studies performed systematic comparisons (e.g., black vs. white water rivers in Laranjeiras et al., 2019) hampering extrapolations. Also, ecologically generalist species seem to have less population differentiation than specialists, as expected for taxa occurring in multiple flooded habitats throughout the Amazon River Basin, compared to those having more restricted distribution ranges (Choueri et al., 2017). However, species‐specific population structure was also observed, again hindering the generalization of such pattern for the majority of lowland bird species (Choueri et al., 2017). In the Amazonian flooded forests, the maintenance of gene flow might inhibit population structure and delay the complete differentiation between new species, even for small‐ranged, specialized taxa (Thom et al., 2018). Clarifying the mechanisms of diversification within Amazonian flooded habitats has also been obscured due to the distinct dispersal ability and degree of habitat specialization of species, resulting in different responses to historical landscape change and multiple independent episodes of specialization to flooded forested environments (Aleixo, 2002; Ribas et al., 2009).

A recent model of the evolution of the Amazon River Basin emphasizes different geological origins for *várzeas* and *igapós* (Andean vs. shield‐sourced) and their resultant western and eastern distribution strongholds, respectively (Bicudo et al., 2019). These features are highlighted to explain a common general pattern of phylogenetic diversity for Amazonian birds, yet mostly influenced by the more numerous *terra firme* species than *várzea* representatives; and the dynamic change in the Amazon River Basin topography during its formation is suggested to have caused spatio‐temporally contrasting diversification events (Bicudo et al., 2019; Pupim et al., 2019 and references therein). Periods of fluvial aggradation resulting in sediment accumulation would have alternated with incisional stages, allowing the cyclic expansion and retraction of inundated habitats, respectively, and the opposite pattern for upland forests (Pupim et al., 2019). Given this instability, species‐specific responses to such changes, often mediated by ecological traits, are expected, which do seem to have occurred during the diversification of Amazonian birds (Bicudo et al., 2019; Choueri et al., 2017; Silva et al., 2019; Thom et al., 2018, 2020). Therefore, more informed descriptions of the ecological and taxonomic diversity of organisms and of the processes maintaining biotic communities within Amazonian lowland forested ecosystems are still needed to fully disclose the effects of the significant topographic, geologic, and climatic changes occurring during the Quaternary (Bicudo et al., 2019; Cheng et al., 2013; Pupim et al., 2019; Ribas & Aleixo, 2019).

The Glossy Antshrike, *Sakesphorus luctuosus*, is a flooded forest specialist distributed in central and southern Amazonia, including the northern edge of the *Cerrado* biome (Zimmer & Isler, 2020). The species is currently divided into two subspecies, *S. l*. *luctuosus* and *S. l*. *araguayae*, which might represent distinct full species since the latter is restricted to Araguaia River, despite the absence of morphological diagnose (Lopes & Gonzaga, 2012; Zimmer & Isler, 2020). A third taxon, *S*. *hagmanni*, has long been considered a junior synonym of *S. l*. *luctuosus*, but it is only known from a single specimen obtained on the south bank of the Amazon River (Taperinha, Pará, Brazil; close to locality 25, Figure 1a), and some authors consider it as *species inquirenda* (Lopes & Gonzaga, 2012; Zimmer & Isler, 2020). Henceforth, we address these three forms as *luctuosus*, *araguayae,* and *hagmanni*, respectively.

Some degree of isolation and population differentiation is expected due to its occurrence in Amazonian riverine islands (Choueri et al., 2017; Cohn‐Haft et al., 2007), and the restriction to clear and white water rivers suggests some adaptation and a likely cohesion between populations occurring in a specific habitat type (Junk et al., 2011; Zimmer & Isler, 2020). However, none of the taxa historically associated with *S*. *luctuosus* is either restricted to river islands or to a particular water type. Glossy antshrikes are not considered threatened, nor major threats have been identified for the species (BirdLife International, 2016; Zimmer & Isler, 2020), but it occurs in highly vulnerable areas, menaced by deforestation, and current and planned construction of several hydropower dams, which directly affect flooded environments (Latrubesse et al., 2017, 2020; Lees et al., 2016).

Here, we investigate genomic diversity within *S*. *luctuosus*, inferring population structure and reconstructing for the first time the spatio‐temporal pattern of diversification within the species. We focus on identifying independent lineages by considering their genetic differentiation levels, divergence time, migration rates and understanding their current and past demographic history. Our ultimate goal is to contribute to a better understanding of the processes shaping diversity within Amazonian *várzeas* and *igapós* and to a more reliable assessment of current biotic diversity associated to Amazonian seasonally flooded habitats, which are under imminent threat due to the construction of hydropower dams along the main Amazonian tributaries.

## 2 MATERIALS AND METHODS

### 2.1 DNA extraction and library construction

Genomic DNA was extracted from tissues of 32 specimens of *S*. *luctuosus* from across its known range, including 5 *araguayae* representatives, and a single specimen collected on a river island located ca. 18 km from the type locality of *hagmanni* on the south bank of the Amazon River (Figure 1a, locality 25; Table S1.1). Because of the geographic proximity to the type locality of *hagmanni*, hereafter we regard this specimen as putative *hagmanni*. We used DNeasy Blood & Tissue kit (Qiagen) for DNA extraction, and Qubit^®^ 2.0 Fluorometer (Life Technologies) to assess quantity and quality of the extracted DNA. Sequence capture and sequencing of Ultra Conserved Elements (hereafter UCEs) were performed according to Faircloth et al. (2012) by RAPiD Genomics (Gainesville, FL, USA). More than 2,300 UCEs and 97 exons were targeted (Harvey et al., 2017; Zucker et al., 2016).

### 2.2 Raw sequences analyses and phylogenetic inference

We followed the PHYLUCE pipeline (Faircloth, 2016, 2017) to first remove adapters, barcodes, and low‐quality sequence regions using Illumiprocessor 2.0.7 (Faircloth, 2013), with the trimming tool Trimmomatic 0.32.1 (Bolger et al., 2014); and to assemble trimmed reads using Trinity (Grabherr et al., 2011). These analyses were performed using default parameters. Contigs were blasted against the probe set of exons and UCEs using phyluce_match_contigs_to_probes. The annotated set was divided by locus, and each was aligned with MAFFT using the scripts phyluce_assembly_get_match_counts, phyluce_assembly_get_fastas_from_match_counts, and phyluce_align_seqcap_align. A complete matrix comprising only loci without missing data was used to obtain a maximum likelihood tree in RAxML (Silvestro & Michalak, 2012; Stamatakis, 2014), following the UCE Phylogenomics tutorial from PHYLUCE pipeline (Faircloth, 2016). Sequences of *S*. *canadensis*, previously recovered as the sister species to *S*. *luctuosus* (Brumfield & Edwards, 2007; see also Harvey et al., 2020), were used as outgroup. Since the resulting phylogenetic tree was poorly resolved (Figure 1b), overall refuting the hypothesis of *S*. *luctuosus* corresponding to a complex of species (Zimmer & Isler, 2020), we proceeded with population genomic analyses for the ingroup. Yet, we identified a highly admixed individual (T22906, locality 25; Figure 1), which could correspond to a *hagmanni* representative due to its origin, close to the type locality of this taxon, but not to its phenotype, which mostly resembled a pure *luctuosus* (data not shown; Lopes & Gonzaga, 2012; Zimmer & Isler, 2020). Thus, to evaluate whether this individual is conspecific with *S*. *luctuosus*, and not a hybrid between *luctuosus* and other *Sakesphorus* or any closely related *Thamnophilus* species (Brumfield & Edwards, 2007; Lopes & Gonzaga, 2012), we used previously published sequences of the mitochondrially encoded NADH dehydrogenase 2 and 3 genes (ND2 and ND3, respectively), representing the most complete dataset available in GenBank for both genera (Brumfield & Edwards, 2007). Since only one representative was available for each species, we added to this dataset one random pure representative of each of the inferred *S*. *luctuosus* populations (Figure 1c) and our sequences for *S*. *canadensis*. We used *Thamnomanes caesius*, *Cymbilaimus lineatus,* and *Frederickena unduligera* as outgroups (Brumfield & Edwards, 2007). To set the best fit model of partition and substitution, we used PartitionFinder (Lanfear et al., 2012). Due to the presence of some missing data for two species, *S*. *melanothorax* and *Thamnophilus multistriatus*, we chose a Bayesian method to infer phylogenetic relationships, as implemented in MrBAYES (Ronquist et al., 2012). We ran 160,000 generations, with a print frequency of 100 and a diagnose frequency of 1,000. The resulting tree was visualized in FigTree (https://github.com/rambaut/figtree/).

### 2.3 Population genomic analyses

We used the seqcap_pop pipeline to extract one biallelic SNP from each locus (Harvey et al., 2016). Briefly, the longest contigs were retrieved from an incomplete matrix, which included all loci identified in all 32 Glossy Antshrike specimens sampled using BWA (Li & Durbin, 2009). These contigs were blasted against the zebra finch genome (*Taeniopygia guttata* v. 3.2.4, NCBI code: GCF_000151805.1), to annotate loci linked to the Z chromosome. These loci were removed from further analyses (Table S2). Then, one biallelic SNP, present in all samples, was randomly chosen from each of the alignments, using the Genome Analyses Tool Kit (McKenna et al., 2010) and VCFTools (Danecek et al., 2011).

To identify *F*_ST_ outliers (i.e., loci likely under selection), we first performed a principal component analysis in the SNPs dataset to set a preliminary population structure using PAST3 (Hammer et al., 2001) and then run BayeScan (Foll & Gaggiotti, 2008). More accurate population structure, genomic assignments, and admixture tests were performed using STRUCTURE 2.3.4 (Pritchard et al., 2000), and the SNPs dataset, without Z‐linked loci and those loci identified as *Fst* outliers. We ran models of one up to six clusters, for 10 iterations of 10^6^ MCMC chains, with a 10^5^ burn in, under default settings. To establish the phylogenetic relationships between the inferred clusters, we used SNAPP implemented in BEAST 2.6 (Bouckaert et al., 2019) and excluded highly admixed individuals regarded as putative hybrids. Mutation rates were automatically calculated by the software, allowing log‐likelihood correction. We ran 5,000,000 generations, storing a tree state every 1,000 generations. Convergence was verified in TRACER 1.7.1 (Rambaut et al., 2018). DensiTree, also implemented in BEAST, was used to visualize the resulting trees, discarding the first 10% as burn‐in (Bouckaert et al., 2019). This analysis was run in the CIPRES Science Gateway V. 3.3 (Miller et al., 2010).

Population differentiation indices were estimated in Arlequin 3.5 running 1,000 permutations to estimate *p*‐values (Excoffier & Lischer, 2011). Genetically admixed individuals (*Q* < 80%) were included and attributed to a population of origin considering a geographic criterion, except for specimen T22906 (putative *hagmanni*), which could not be attributed to either a geographic or a genetic population (Figure 1).

The rangeExpansion package in R (Peter & Slatkin, 2013, 2015) was used to infer the approximate origin and most likely directionality of expansion, also using the SNPs dataset. Both single origin and multiple origins were tested. In the case of multiple origins, population groups were delimited based on geographic location, genetic assignment, and water type (Figure 1). Admixed individuals were also included. Moreover, due to analytical constraints, two sets of areas were tested, since each population must be represented by more than two individuals. We tested a set representing the most likely population structure and considering the western population (W, located west of the Madeira River), the southeastern (SE, loosely delimited by Tapajós and Xingu rivers), and the *Cerrado* population (CE, occurring in the *Cerrado* gallery forests, upper Araguaia River). The second set comprised all individuals assigned as: (a) W+ highly introgressed T22906; (b) SE+N (including specimens from the Paru River margins); and (c) CE populations. Overall, our range expansion scenarios estimated with rangeExpansion need to be interpreted with caution, since the movement of glossy antshrikes is essentially restricted to river channels (riverine forests along banks and islands). However, the demographic models implemented in rangeExpansion consider uniform probabilities of dispersal throughout the species range (Peter & Slatkin, 2013, 2015), which in the case of the glossy antshrikes would imply in dispersal across interfluvial areas covered mostly by upland *terra firme* forest. Even though glossy antshrikes also occur along margins of small tributaries deep into some Amazonian interfluves that are not subject to extensive flooding (Zimmer & Isler, 2020; Figure S1; A. Aleixo, personal observation), dispersal could be ultimately constrained by the presence of large tributaries (see Section 3), and therefore, a uniform dispersal prior across the entire species range may not apply.

To estimate relative migration and effective population sizes for each of the four geographic and genetically supported populations in our data set, and infer differentiation times between them, a new complete matrix was generated, again excluding specimen T22906. We identified and selected 132 loci with 544 informative sites using phyluce_align_get_informative_sites to be used in a multi‐species coalescent analysis implemented in G‐PhoCS (Gronau et al., 2011). Following the results from previous analyses (Figure 1 and Table 1), we set the phylogenetic relationships as (((N, SE), W), CE) and considered recent splits (time of divergence estimated as *τ *= *Tμ*/*g*; where *μ* represents the mutation rate and *g* the generation time), setting priors for *τ* gamma distribution as *α* = 2 and *β* = 2,000. Nonetheless, a model considering old divergences was also included (*α* = 1 and *β* = 10). Different prior sets to model effective population sizes were set either considering small or large sizes (effective population size estimated as *θ* = 4*Neμ*), *α* = 2 and *β* = 2,000 and *α* = 1 and *β* = 10, respectively. Migration (*M* = *m*/*μ*) was set to *α* = 0.002 and *β* = 0.00001. Each analysis was run during 10^6^ generations after a burn in of 10^4^ generations, with finetune parameters. TRACER was used to summarize statistics for each run and also to check for convergence (Rambaut et al., 2018). To discuss the calibrated estimates, we assumed a mutation rate (*μ*) of 6.75 × 10^−10^ mutations/site/year estimated for UCEs based on comparisons across the Passeriformes phylogeny and its sister group (Psittaciformes) using complete genomes and fossil‐dated divergence ages (Winker et al., 2018). Faster (*μ* = 3.0 × 10^−9^ mutations/site; Smith et al., 2014) and slower rates (μ = 1.1 × 10^−12^ mutations/site; Zucker et al., 2016) have been reported for UCEs +exons datasets in other passerines. We considered the Winker et al. (2018) a more robust estimate than the others since it was based on comparisons using complete genomes and fossil‐dated divergence ages across the Passerine lineage and its sister group (Psittaciformes). In contrast, the other UCE calibrations mentioned above were inferred indirectly by comparison to estimates of substitution rates of the ND2 mitochondrial gene (Smith et al., 2014; Zucker et al., 2016), which are subject to higher error rates. Finally, a specific generation time (*g*) of 2.81 year estimated for the Glossy Antshrike was considered (Bird et al., 2020), but a shorter alternative generation time was also used (*g* = 1 year, described for the sister species *S*. *canadensis*; del Hoyo et al., 2020).

**TABLE 1:** Genetic differentiation (*F*_ST_) estimated for the Glossy Antshrike *Sakesphorus luctuosus* populations inferred in this study (lower diagonal) and respective *p*‐values (upper diagonal; ≤.05)

	W	CE	SE	N
Western Amazon W		0.000	0.000	0.010
*Cerrado* CE	0.27		0.000	0.045
Southeastern Amazonia SE	0.12	0.29		0.015
Northern Amazonia N	0.18	0.37	0.16	

Note

Western Amazonia population occurs in white water rivers, whereas all the others are located in forests flooded by clear waters (Junk et al., 2011).

Abbreviations: CE, *Cerrado*; N, northern Amazon River; SE, southeastern Amazonia; W, western Amazonia.

Last, we selected the 30 UCE loci with more informative sites for each population separately to estimate their demographic histories under an extended Bayesian skyline plot approach as implemented in BEAST 2 (Bouckaert et al., 2014; Heled & Drummond, 2008). In this case, only genetically “pure” individuals (*Q* > 0.80; Figure 1c) were used. The most appropriate mutation models were set to be estimated by BEAST Model Test. A strict clock with uniform distribution was chosen, using the same mutation rate detailed above as reference, but allowing the program to estimate this variable to account for the uncertainty of this prior. A chain length of at least 10^8^ generations was run for each population. Again, convergence of the runs was verified in TRACER (Rambaut et al., 2018).

## 3 RESULTS

After quality control and trimming (Table S1.2), we obtained a dataset of 2,372 UCEs and exons (incomplete matrix), from which 242 loci were recovered for all samples, including the outgroup (complete matrix). Phylogenetic analysis using UCEs recovered *araguayae* specimens clustered together in a clade with 100% of support, but all the other relationships within the species were not resolved (Figure 1b). The phylogeny based on mitochondrial DNA supported the monophyly of *S*. *luctuosus*, clustering *luctuosus*, *araguayae,* and the putative *hagmanni* representative in a statistically well‐supported clade (PP = 1; Figure S2).

To further explore population structure, we recovered 1,070 SNPs from an incomplete matrix (i.e., all 1,070 loci identified in the ingroup data set). Of these, 61 loci were likely linked to the Z chromosome (Table S2) and one locus was probably under selective pressure (Figure S2). A final data set of 1,008 SNPs was used to perform population structure analyses. STRUCTURE supported the existence of four clusters (Table S2; Figure 1c). According to this analysis, the most differentiated population occurs in the southeasternmost portion of the species distribution in the upper Araguaia River in the *Cerrado* wetlands (Figure 1). Several admixed individuals were identified, with levels of ancestry correlating with their geographic origin (Figure 1a,c). Specimen T22906 had an admixed genome represented by all the three Amazonian populations observed (Figure 1a,c). This admixed composition is also observable in models considering superior *k* values (*k* = 5 and *k* = 6; data not shown). *F*_ST_ values among populations were all statistically significant (*p* < .05) and superior to 0.12 (Table 1).

**FIGURE 2:**
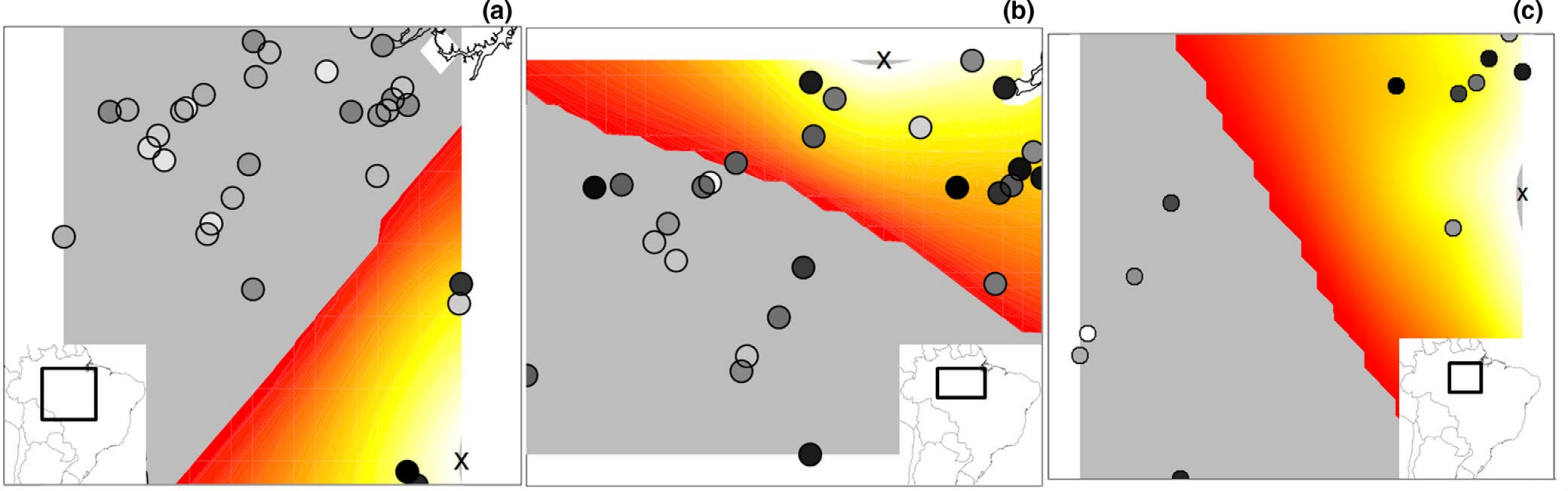
Putative origin of expansion considering (a) the whole dataset or (b) excluding samples from the flooded forests within *Cerrado*. While testing for multiple origins, isolation by distance was only rejected for the southeastern Amazonian population (c). The color gradient indicates the purported origin (in yellow) and front (in red) of expansion

Considering the full dataset or excluding *araguayae* (as this seems a more differentiated population), a likely significant deviation from isolation by distance equilibrium was recovered across the range of *S*. *luctuosus*, with small strength of the founder effect, and large founder distances (>25 km; Table S4). A putative origin of expansion might have been located in the *araguayae* area of distribution for the full data set, or further north when excluding this population (Figure 2a,b; Table S4). When accounting for the possibility of multiple origins of expansion, an isolation by distance scenario was only rejected for the southeastern Amazonian population; so, only results for this population are reported (Figure 2c; Table S4). In this case, effective founder distances were estimated to be smaller than 15 km (Table S4). Putative origin of expansion was likely in the easternmost limit of distribution of this population (Figure 2c).

**FIGURE 3:**
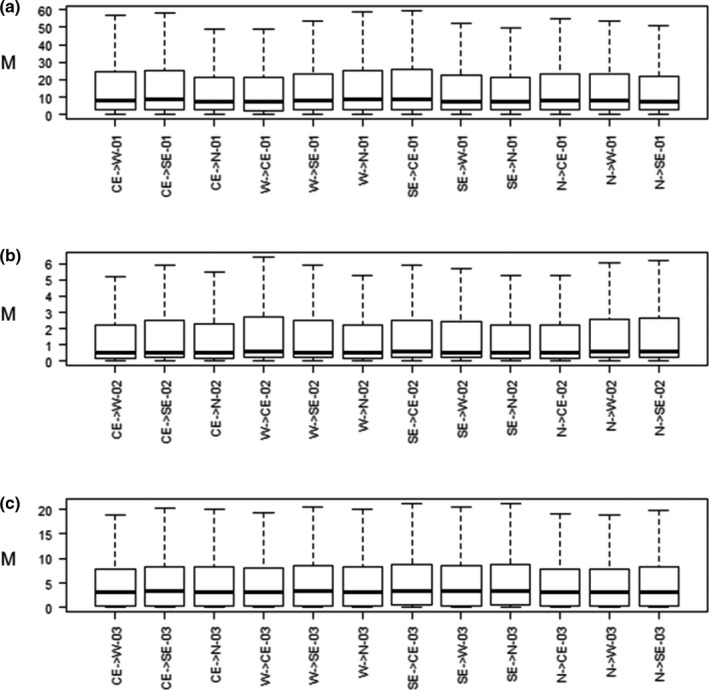
Raw migration estimates (*M* = *m*/*μ*) for the Glossy Antshrike *Sakesphorus luctuosus* populations under different models of evolution, considering recent divergences with (a) small or (b) large effective population sizes, or (c) old divergences with large effective population sizes. Populations: western Amazonia (W), southeastern Amazonia (SE), northern Amazon River (N) and in the flooded forests within *Cerrado* (CE). Outliers were omitted for simplicity. Note that *y*‐axes have different scales

**FIGURE 4:**
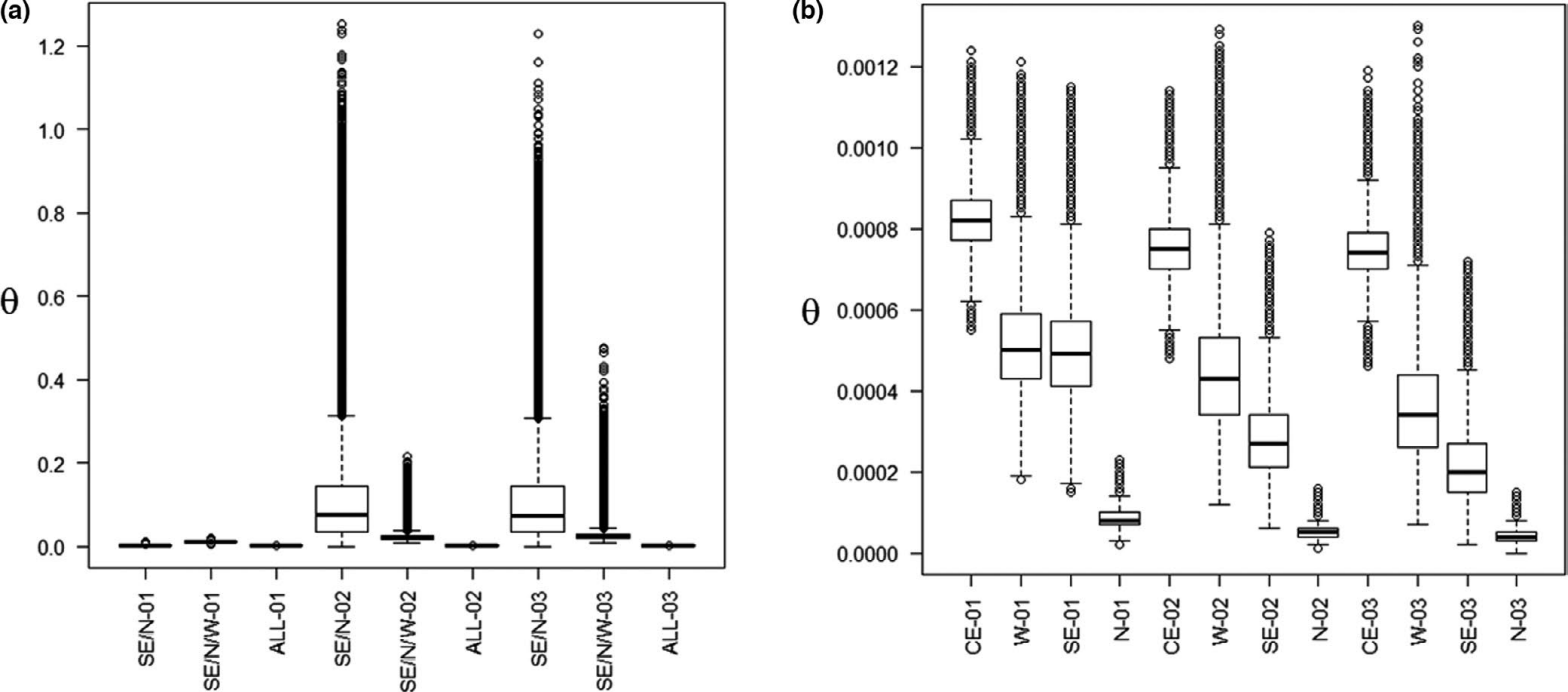
Raw demographic parameters estimated for the Glossy Antshrike *Sakesphorus luctuosus* populations under different models of evolution. (a) Ancestral and (b) modern *θ* = 4*Neμ*, effective population size estimates. Models of evolution consider recent divergences with small (01) or large effective population sizes (02), or old divergences with large effective population sizes (03). Populations: western Amazon (W), southeastern Amazon (SE), northern Amazon River (N) and in the flooded forests within *Cerrado* (CE). Note that y‐axes have different scales

The dataset used to estimate relative migration levels and time of differentiation for the Glossy Antshrike populations comprised 132 loci with 98,509 bp and 544 informative sites (between 2 and 13 per locus). Overall, mean levels of migration were similar between pairs of populations for any of the models considered, but confidence intervals did vary between estimates, with multiple statistical outliers (Figure 3). Population splits seem to have occurred during the last 1.24 mya (C.I.:1.04–1.45 mya; or 444 kya C.I.:370–518‐, if *g* = 1 year), with the most recent events at 125 kya (C.I.:83–291 kya; or 44 kya C.I.:30–104) and 83 kya (C.I.:42–208 kya; or 30 kya C.I.:15–74; Figures 1d, S4), respectively. Ancestral effective population sizes seem to have increased since the last common ancestral population (Figure 4a). Nonetheless, uncertainty for models considering large population sizes seems to have affected the results, given the broad presence of statistical outliers (Figure 4a). Modern effective sizes seem to be higher for the population within *Cerrado* wetlands (*araguayae*), followed by southeastern and western populations and smaller for the northern population (Figure 4b).

**FIGURE 5:**
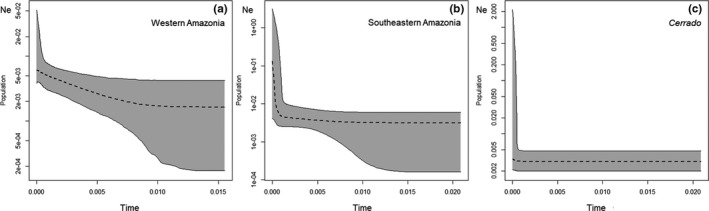
Demographic population trends for each of the populations observed from our data. Admixed individuals (*Q* < 80%) were excluded from these analyses. The population from the north of the Amazon River was not considered since only two specimens were sampled. Dashed lines represent the median effective population size, and the colored area represents the 95% highest posterior density interval. *Y*‐axes are in a logarithmic scale, and *x*‐axes represent time in million years

To estimate the historical and current demographic trends for each population, we used 30 loci with 235, 164, and 109 informative sites for populations in western Amazonia, southeastern Amazonia, and flooded forests within *Cerrado*, respectively. The population from northern Amazonia was represented by an insufficient number of individuals to be included in this analysis (*n* = 2). The Glossy Antshrike has experienced very recent (Holocene) population expansion within western (Figure 5a) and southeastern Amazonia (Figure 5b), while the population from *Cerrado* flooded forests has probably been stable (Figure 5c).

## 4 DISCUSSION

### 4.1 Taxonomic implications

Both *araguayae* and *hagmanni* were once considered junior synonyms of *S. l*. *luctuosus* and therefore undistinguishable from it (Lopes & Gonzaga, 2012; Zimmer & Isler, 2020). However, a comprehensive analysis of the distribution and phenotypic variation in such broadly defined *S*. *luctuosus* (including *hagmanni* and *araguayae*), concluded that *hagmanni* was distinct enough to warrant formal taxonomic recognition, although the lack of additional specimens prevented any conclusion concerning its alternative ranking as species or subspecies (Lopes & Gonzaga, 2012). With respect to *araguayae*, this same study concluded that it should represent the end of a cline of morphological variation within S. *luctuosus* as a whole (Lopes & Gonzaga, 2012). Our molecular results support *S*. *luctuosus* as a single monophyletic group, with both *araguayae* and putative *hagmanni* clustering within *S*. *luctuosus* according to both phylogenetic analyses performed (Figure 1b—UCEs and exons, and Figure S2—ND2 and ND3). Despite all *araguayae* specimens grouping together in a highly supported clade in the UCEs phylogeny, relationships of *araguayae* with other *S*. *luctuosus* populations were not resolved (Figures 1b,d, S2). Moreover, overall levels of gene flow seem to be similar among all populations recovered (Figure 3a). Such level of admixture also coincides with morphological data from the most recent taxonomic revision on *S*. *luctuosus*, which identified a high degree of phenotypic polymorphism throughout the species' range (Lopes & Gonzaga, 2012). Accordingly, scenarios modeling recent divergences (Figure 3a) presented higher migration rates than those accounting for old splits (Figure 3c). Calibrated time estimates for population differentiation events set the first split back to the Middle Pleistocene at the latest (Figures 1d, S4).

On the other hand, contrary to the expectations of Lopes and Gonzaga (2012), admixture zones seem to be overall well‐delimited throughout *S*. *luctuosus* range, with no support for a latitudinal cline. Instead, our results support the presence of four geographically structured populations (Figure 1a,c; Table 1), revealing a higher phyletic diversity than that suggested by current taxonomy (Zimmer & Isler, 2020). Due to morphological dissimilarities between our putative *hagmanni* and the taxon´s actual holotype (data not shown), the status of *S*. *hagmanni* still needs further evaluation (Lopes & Gonzaga, 2012). However, it is noteworthy that our lone specimen coming from nearby (ca. 18 km) *S*. *hagmanni* type locality, was the most admixed specimen among all specimens sequenced (Figure 1c), suggesting that rather than an “aberrant form” (Lopes & Gonzaga, 2012), *hagmanni* might correspond to a highly admixed population originating from the introgression of three distinct phylogroups of *S*. *luctuosus* (N, SE, and W; Figure 1a,c; Table 1). Interestingly, both the *hagmanni* type locality and locality 25 (where our putative *hagmanni* was obtained) are located on the contact zone among the N, SE, and W phylogroups of S. *luctuosus* (Figure 1b), reinforcing the notion that this taxon represents a locally highly introgressed population (see below).

Populations attributed to the *luctuosus* subspecies, that is, northern, southeastern and western, are in fact more similar to each other than to the population occurring in the *Cerrado* flooded gallery forests (or *araguayae* population; Table 1; Figure 1a,c). While the putative factors driving population differentiation in the Glossy Antshrike are discussed below, we conclude that *araguayae* represents a geographically and genetically distinct population of *S*. *luctuosus*, which is potentially threatened due to loss of floodplain habitat along the Araguaia/Tocantins River (Coe et al., 2011; Latrubesse et al., 2017, 2020; Lininger & Latrubesse, 2016). Moreover, nominate *luctuosus* includes at least three distinct populations (western, northern, and southeastern).

### 4.2 Spatio‐temporal factors affecting the Glossy Antshrike population structure

Population differentiation in the Glossy Antshrike partially correlates with geography. Overall, contiguous populations are more similar than those farther apart (e.g., western vs. southeastern *F*_ST_ = 0.12 and western vs. *araguayae F*_ST_ = 0.27). Yet, a well‐known distribution gap within the middle and upper Xingu River (see Figure S1 for location of our and gbif.org records and the review by Lopes & Gonzaga, 2012: Figure 1) coincides with the higher genetic differentiation indices observed between *araguayae* and the remaining populations within the *luctuosus* group (*F*_ST_ ≥ 0.27), suggesting that isolation might have contributed to population differentiation. In this region, the flooding period is limited to less than half a year and elevation increases to more than 300 m (Figure S1). These features combined restrict the extension of flooded areas in the region, limiting the area available for the species. Therefore, altitude, and how it correlates with the extent of seasonally flooded forests in southern Amazonia, seems to be a preponderant factor influencing the Glossy Antshrike distribution and population structure. Similarly, the southern tributaries of the Amazon River, Xingu and Tapajós, are particularly influenced by the tidal cycles influencing sedimentary deposition in the *ria* lakes (i.e., drowned river valleys) at the mouth of these rivers (Fricke et al., 2017). These *ria* lakes are currently more than 100 km in length and 10 km in width, without islands (Fricke et al., 2017), likely forming a barrier isolating populations of floodplain‐associated species within each tributary's subbasin. The contact zone between different populations we detected along the Amazon River is spatially concordant with contact zones detected among populations of other birds associated with flooded environments (Thom et al., 2018, 2020), reinforcing the hypothesis that *hagmanni* represents a highly admixed population. Nonetheless, we cannot rule out that local adaptation might also have played some role in population differentiation in *S*. *luctuosus*, as also proposed by Thom et al. (2018) for another floodplain‐associated antbird species. *Sakesphorus luctuosus* is absent from vegetation flooded by low fertility, black water rivers (Zimmer & Isler, 2020), which are normally occupied by its sister species *S*. *canadensis* (A. Aleixo, personal observation), and all populations sampled can be associated with clear or white water types (Figure 1b). White waters feed complex ecosystems, with high trees and a mosaic of niches, while clear water river floodplains harbor a mixture of species, presenting intermediate levels of fertility (Junk et al., 2011). Compared to black water habitats, white and clear water forests might provide more resources to support the species' occurrence, but can be distinct enough between them to influence population differentiation (see Beheregaray et al., 2015; Cook et al., 2012). However, we did not assess adaptive genetic variation in *S*. *luctuosus*; and divergence between southeastern (clear water) and western (white water) populations is the smallest observed among all populations of *S*. *luctuosus* (about 1%: Table 1), a differentiation compatible with either local adaptation or intermittent lack of connectivity along the lower Tapajós (Cohn‐Haft et al., 2007; Moraes et al., 2016).

Nonetheless, connection of eastern *igapós* with white water *várzea* habitats in western Amazonia might be recent (Bicudo et al., 2019; Pupim et al., 2019), as well as the differentiation between southeastern and western populations of the Glossy Antshrike (about 125 kya; or 44 kya if assuming *g* = 1 year; Figures 1d, S4); the genetic patterns observed seem to result from an expansion event, likely toward the west, as suggested by phylogenetic analysis (Figure 1b,d) and rangeExpansion results (Figure 2), contradicting the general expectation that most floodplain adapted taxa would have more stable and long‐lasting populations in the larger western *várzeas* (Aleixo & Rossetti, 2007; Pupim et al., 2019), and adding to the hypothesis of ongoing adaptation of some floodplain species to white waters (Thom et al., 2018). In contrast, the *araguayae* population seems to have been demographically stable (Figure 5c), while recent bursts of expansion of southeastern and western populations seem to have been simultaneous (Figure 5a,b). Connection between clear water (east) and white water flooded forests (west) was periodic since the Plio‐Pleistocene (Bicudo et al., 2019) and, due to the dynamic formation of the Amazon River Basin, this connection went through cycles, with the last establishment of current connectivity occurring along the last 45 ky (Pupim et al., 2019). Moreover, gallery and flooded forests within *Cerrado*, inhabited by the *araguayae* population, coincide with geologically more stable areas (Bicudo et al., 2019; Pupim et al., 2019). Thus, this region could have held ancestral populations of the Glossy Antshrike that were only able to demographically and spatially expand toward the west during one of such periods of connection between western and eastern Amazonian drainages since the Middle Pleistocene. A recent colonization of the western white water habitats, associated with the presence of *S*. *canadensis* (sister species) west of Purus River and so likely imposing ecologic exclusion, would also explain the western limit of distribution for the Glossy Antshrike.

### 4.3 Conclusion and implications for the study of Amazonian flooded forests

Taxonomic uncertainties and biased sampling influence the current knowledge on the diversity and distribution of Amazonian taxa, hindering the inference of the evolutionary processes shaping regional biodiversity (Hortal et al., 2015). Furthermore, species‐specific ecological, behavioral, and life‐history traits result in idiosyncratic responses to such processes further hampering the disclosure of common factors, particularly at micro‐evolutionary, intraspecific scales (Silva et al., 2019; Smith et al., 2014). All these shortfalls combined have been limiting the study of birds associated with Amazonian flooded forests (Aleixo, 2006; Ribas et al., 2009; Thom et al., 2018, 2020). The Glossy Antshrike is no exception and we had to do some compromises: Some areas were not represented by tissue samples (e.g., lower Tocantins River; Figure S1), but a continuity of favorable habitat has been reported along Araguaia and Tocantins rivers (Lopes & Gonzaga, 2012). Also, breeding behavior is poorly known, and so generation time was based on estimates rather than empirical data (Bird et al., 2020; Zimmer & Isler, 2020). However, we clearly show that *Sakesphorus luctuosus* is not a complex of species and suggest it is more closely associated with clear water habitats than previously thought (Zimmer & Isler, 2020). In a scenario of recent connection and origin of modern flooded forested habitats (Bicudo et al., 2019; Pupim et al., 2019), our results suggest that habitat availability might be one of the most relevant factors shaping current population structure of flooded forest birds in southeastern Amazonia; with species recently colonizing and perhaps still adapting to the western *várzeas*. Such directionality adds to the general pattern being described for lowland birds occurring in flooded forests (Thom et al., 2018, 2020). In part, our data agree with initial predictions postulated for specialist species, as the simplest model of vicariant isolation (due to the absence of available habitat resulting in intermittent interruptions of connectivity among populations) cannot be ruled out as a contributing diversifying factor (Aleixo, 2006; Cohn‐Haft et al., 2007; Ribas et al., 2009; Thom et al., 2018). However, we emphasize that the role of adaptation and interspecific competition should be further investigated, particularly when associated with distinct water types (Laranjeiras et al., 2019, but see also Thom et al., 2018), and their corresponding dissimilar histories (see Bicudo et al., 2019, although temporal scales might be different from ours).

Deforestation, climate change, and other anthropogenic perturbations to the Amazonian flooded and upland ecosystems are neither distributed evenly across the basin nor their effects are similar among distinct habitat types (Anderson et al., 2018; Latrubesse et al., 2010, 2017; Lees et al., 2016; Renó et al., 2016). For instance, the so‐called “Dam Environmental Vulnerability Index” is higher in the distribution gap separating the *araguayae* population from the other populations and within the contact zone between southeastern and western Glossy Antshrike populations (Figure S1; Latrubesse et al., 2017). Due to dam construction, the permanent drought in a region known as Volta Grande do Xingu will likely further restrict the distribution and isolate the southeastern population in the near future, decreasing considerably connectivity between flooded environments. In a context in which panmixia is rejected, and although populations seem to be expanding (Figure 5), geographically distinct threat levels might further promote isolation and differentiation of such demes, interrupting the potential natural adaptive processes and accelerating the loss of unique biodiversity components.

## CONFLICT OF INTEREST

All authors declare no competing financial, professional, or personal interests related to the analyses, results, and interpretations presented in this manuscript.

## AUTHOR CONTRIBUTIONS

**Sofia Marques Silva:** Conceptualization (equal); Data curation (lead); Formal analysis (lead); Investigation (equal); Methodology (equal); Project administration (lead); Software (lead); Validation (equal); Visualization (lead); Writing‐original draft (lead); Writing‐review & editing (equal). **Camila C. Ribas:** Conceptualization (equal); Data curation (supporting); Formal analysis (supporting); Funding acquisition (lead); Investigation (equal); Methodology (equal); Project administration (supporting); Resources (equal); Supervision (supporting); Validation (equal); Writing‐review & editing (supporting). **Alexandre Aleixo:** Conceptualization (equal); Formal analysis (supporting); Funding acquisition (supporting); Investigation (equal); Methodology (equal); Project administration (supporting); Resources (equal); Supervision (lead); Validation (equal); Writing‐review & editing (equal).

## Supporting information

Supplementary Material

## Data Availability

Raw Illumina sequences: Geanbank BioProject ID PRJNA703443. Complete matrix: Dryad https://doi.org/10.5061/dryad.sxksn032q. Final SNPs dataset: Dryad https://doi.org/10.5061/dryad.w9ghx3fp6.
