# Supplementary material for: Recent population differentiation in the habitat specialist Glossy Antshrike (Aves: Thamnophilidae) across Amazonian seasonally flooded forests
Source: Ecol Evol. 2021 Jul 28;11(17):11826–38. doi: 10.1002/ece3.7951 (PMC8427616; doi:10.1002/ece3.7951)
Supplement: Supplementary file 1 — Supplementary Material [file ECE3-11-11826-s001.docx]

**Supplementary Material**

**Recent population differentiation in the habitat specialist Glossy Antshrike (Aves: Thamnophilidae) across Amazonian seasonally flooded forests**

Sofia Marques Silva^1,2^; Camila C. Ribas^3^; Alexandre Aleixo^2,4,*^

^1^ Research Centre in Biodiversity and Genetic Resources, CIBIO/InBIO, R. Padre Armando Quintas 7, 4485-661 Vairão, Portugal

^2^ Museu Paraense Emílio Goeldi, Department of Zoology, 66040-170 Belém, Pará, Brazil.

^3^ Instituto Nacional de Pesquisas da Amazônia, INPA, Campus II, Av. André Araújo 2936, 69060-000 Manaus, Amazonas, Brazil

^4^ Finnish Museum of Natural History, University of Helsinki, 00014 Helsinki, Finland

^*^ Corresponding author; e-mail: alexandre.aleixo@helsinki.fi

This document includes:

Figure S1. Additional maps depicting mean annual flooding months for the Amazon River Basin and altitude for the region.

Figure S2. Phylogenetic relationships among *Sakesphorus luctuosus* inferred populations and *Thamnophilus* species estimated by Bayesian inference for mitochondrial loci.

Figure S3. *Fst* outliers’ analyses.

FIGURE S4. Divergence time estimates.

Table S1. Details on the samples used in this study and respective DNA processing.

Table S2. Putative Z-linked loci.

Table S3. Summary from Bayesian clustering analyses.

Table S4. Origins of expansion inferred from models considering one or multiple events.


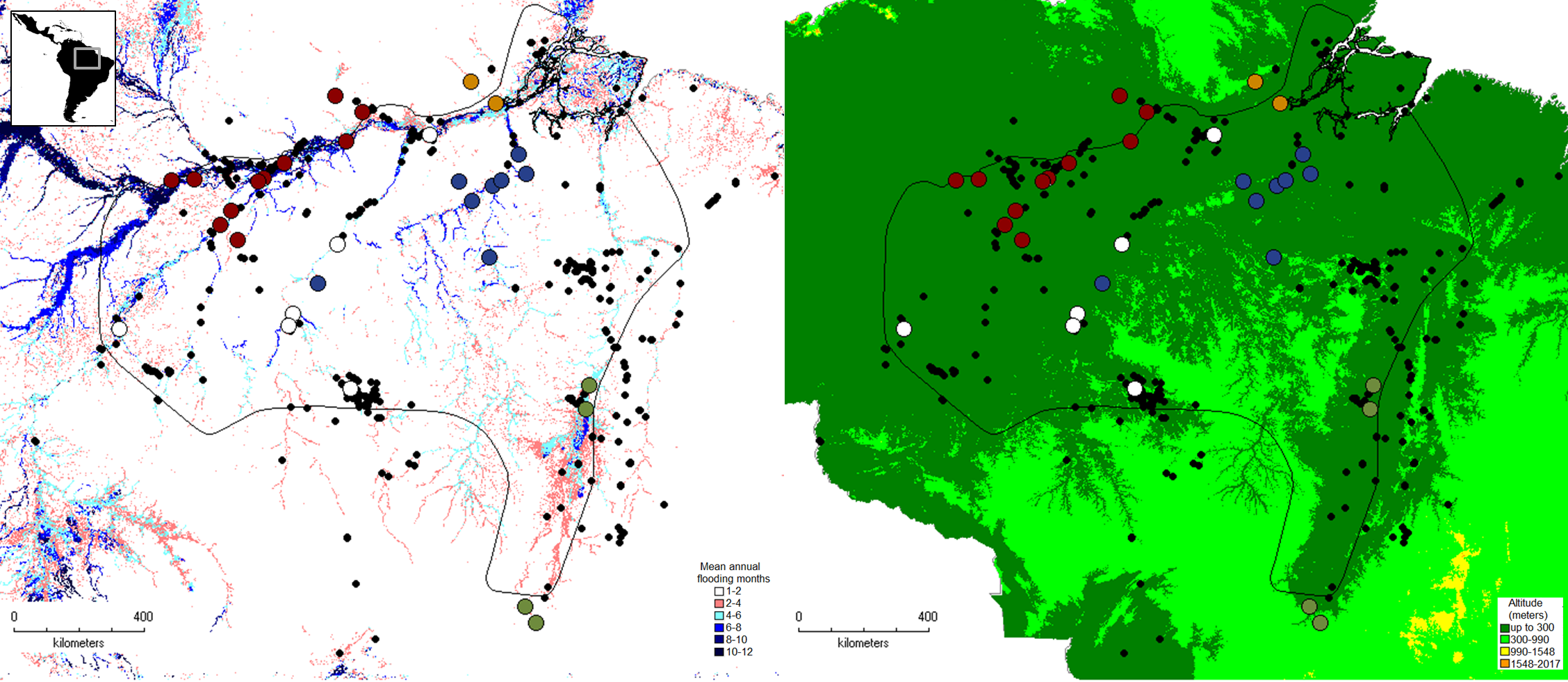


**Figure S1.** The geographic distribution of the Glossy Antshrike *Sakesphorus luctuosus*, depicted by the delimited area (BirdLife International, 2016), is compared with the population structure found in our study (bigger colored dots, corresponding with Figure 1; highly admixed individuals were intentionally left in white for emphasis) and observations retrieved from the Global Biodiversity Information Facility database (smaller black dots) ([www.gbif.org](http://www.gbif.org); last accessed 21.11.2019; see also Lopes & Gonzaga, 2012): Figure 1 and www.ebird.org). Left map: mean annual flooding months for the Amazon River Basin (adapted from Miguez‐Macho & Fan, 2012). Right map: altitude for the area of distribution of the species (www.diva-gis.org; last accessed 21.11.2019).


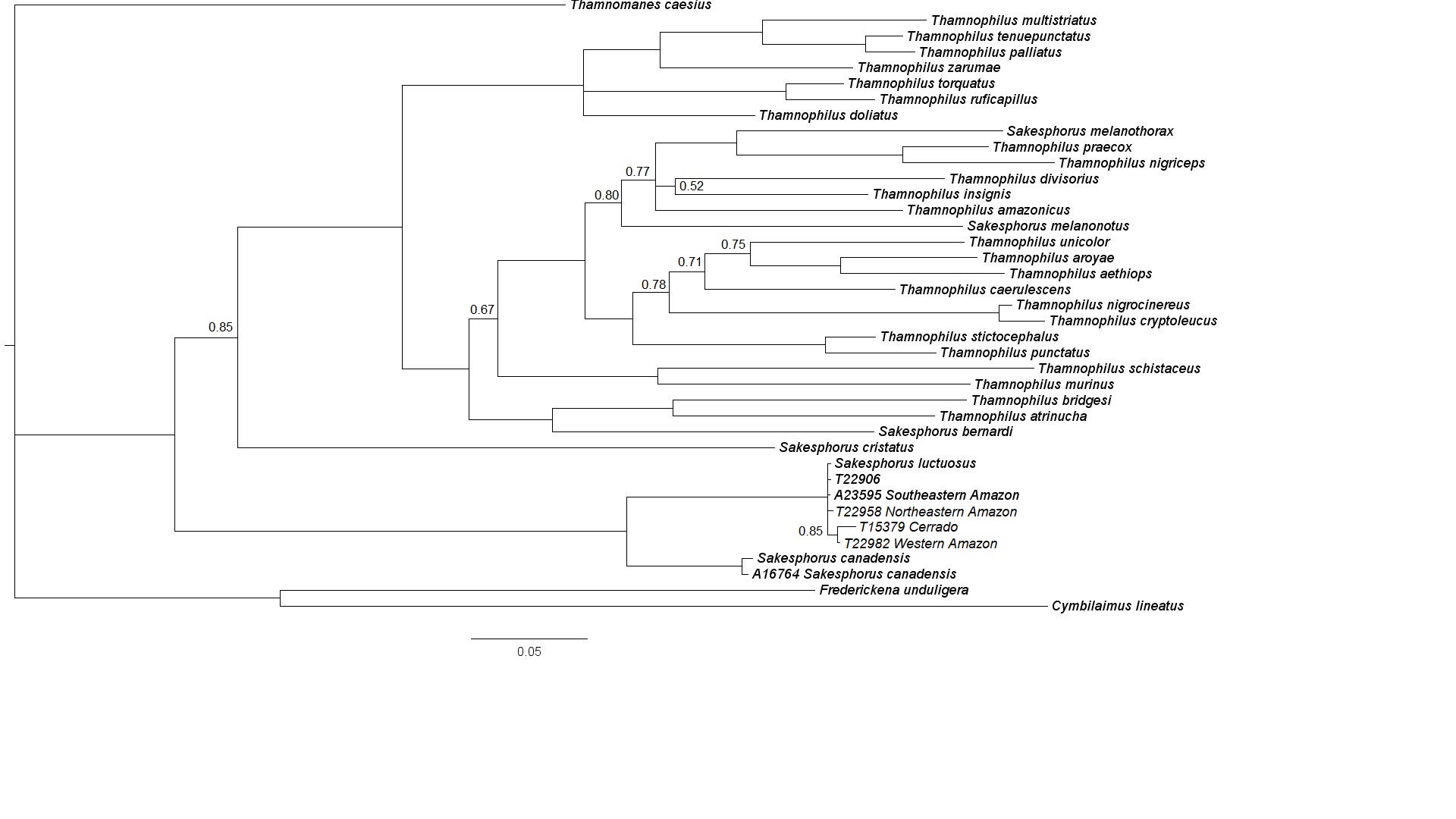
**Figure S2.** Phylogenetic relationships among *Sakesphorus luctuosus* inferred populations and *Sakesphorus / Thamnophilus* species estimated by Bayesian inference (BI) for mitochondrial loci (see methods for details). *Thamnomanes caesius*, *Cymbilaimus lineatus* and *Frederickena unduligera* were used as outgroups. Only posterior probabilities below 0.97 are depicted. T22906 putatively represents *S. hagmanni* due to its geographical proximity to this taxon´s type locality*.* Populations were named after both geographic provenance and based on analyses of population structure (see Figure 1).


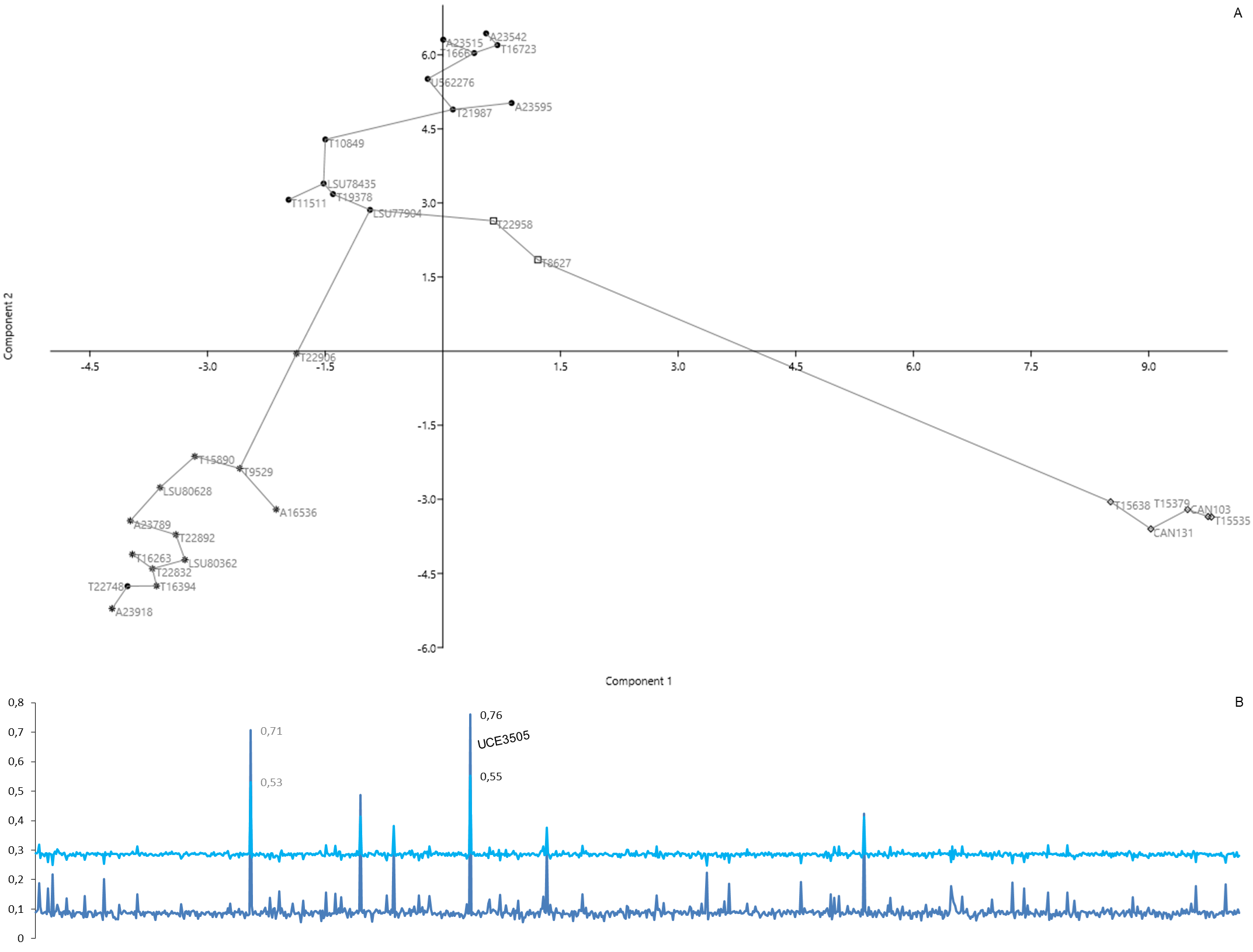


Figure S3. (A) Scatter plot from principal component analysis depicting the minimum spanning tree for the 32 samples from *Sakesphorus luctuosus* analyzed. From the four groups visually identified (represented by stars, dots, squares and losangs, respectively), we estimated (B) *Fst* values (light blue) and respective posterior probability (dark blue) under models of differentiation including selection for each of the 1070 SNPs randomly chosen. PP >0.75 suggests UCE3505 is under selective pressure (Foll & Gaggiotti, 2008).


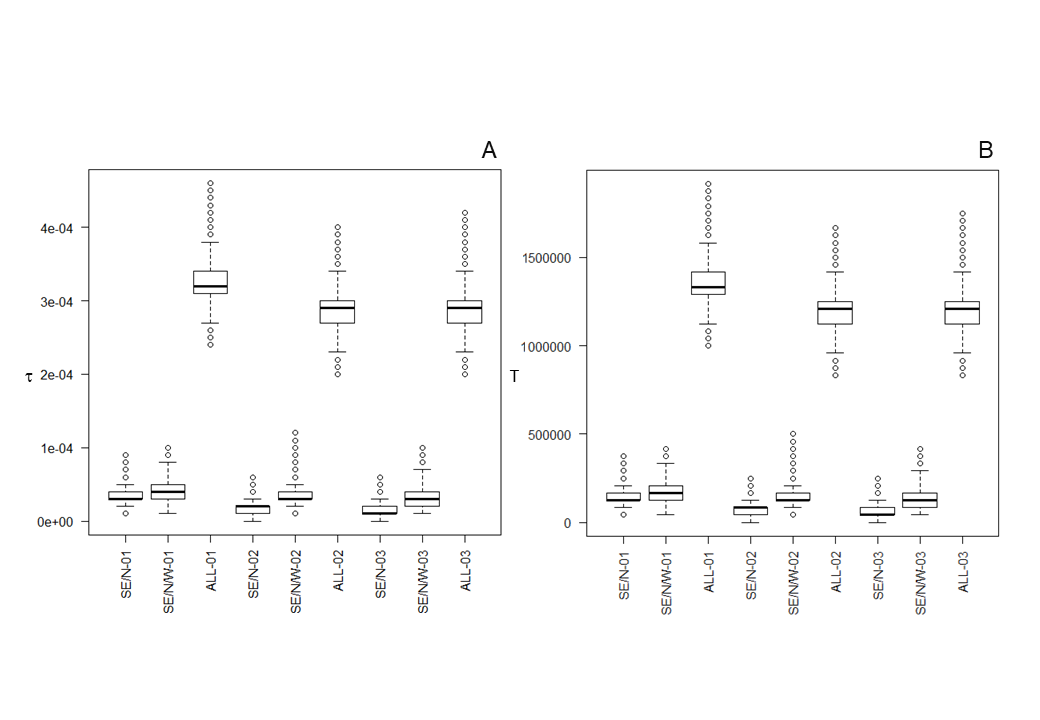
Figure S4. Divergence time estimates for the Glossy Antshrike *Sakesphorus luctuosus* populations under different models of evolution, considering recent divergences with small (01) or large effective population sizes (02), or old divergences with large effective population sizes (03). A) Raw estimates represented as τ=Tμ/g, and B) time of divergence (T) considering a mutation rate (μ) of 6.75x10^-10^ mutations/siteyear ( Winker, Glenn, & Faircloth, 2018) and a generation time (g) of 2.81 years (Bird et al., 2020). Populations: western Amazon (W), southeastern Amazon (SE), northern Amazon River (N) and in the flooded forests within *Cerrado* (CE).

Table S1.1 Samples from *Sakesphorus luctuosus* and *S. canadensis* (*) sequenced in this study. Localities are numbered as in Figure 1; INPA: Instituto Nacional de Pesquisas da Amazônia, MPEG: Museu Paraense Emílio Goeldi, LSUMNS: Louisiana State University, USNM: National Museum of Natural History.

| **Tissue / Voucher #** | **Origin** | | | **Institution** |
| --- | --- | --- | --- | --- |
|  | **Locality** | **Lat** | **Long** |  |
| A23918 | 1. Paraná dos Mundurucus; ca. 45km SW Manacupurú; Brazil | -3.63867 | -60.87583 | INPA |
| T16394 | 2. Autazes; Uricurituba; Ilha; Brazil | -3.5919 | -58.9431 | MPEG |
| A23789 | 3. Boca do Purus; Rio Purus right margin/ Rio Solimões right margin; Brazil | -3.70366 | -61.4585 | INPA |
| T22892 | 4. Parintins; Rio Amazonas left margin; Brazil | -2.5797 | -56.6792 | MPEG |
| T22832 | 5. Itacoatiara; Rio Amazonas; Ilha do Risco; Brazil | -3.1586 | -58.3703 | MPEG |
| T22748 | 6. Borba; Rio Madeira; Ilha do Mandi; Brazil | -4.4803 | -59.8617 | MPEG |
| T16263 | 7. Novo Aripuanã; Prainha; W Bank Rio Madeira; Brazil | -4.8897 | -60.1597 | MPEG |
| 80362 | 8. Rosarinho island in Rio Madeira in front of town of Rosarinho; Amazonas; Brazil | -3.6837 | -59.0937 | LSUMNS |
| A16536 | 9. Rebio Trombetas; Rio Trombetas left margin; Brazil | -1.4167 | -56.7500 | INPA |
| T9529 | 10. Oriximiná; Lake Sapucuá; Brazil | -1.7658 | -56.2267 | MPEG |
| 80628 | 11. Island in Rio Acari Mouth; Amazonas; Brazil | -5.2911 | -59.6758 | LSUMNS |
| T15890 | 12. Humaitá; Mirari; W Bank Rio Madeira; Brazil | -7.7758 | -62.9414 | MPEG |
| 77904 | 13. Barra de Sao Manuel; Amazonas; Brazil | -7.3600 | -58.1386 | LSUMNS |
| T19378 | 14. Itaituba;Rio Tapajós right margin; Rio Rato; Brazil | -5.4306 | -56.9044 | MPEG |
| 78435 | 15. Rio Juruena; Amazonas; Brazil | -7.6853 | -58.2528 | LSUMNS |
| T11511 | 16. Paranaíta; Rio Teles Pires; Brazil | -9.5028 | -56.7592 | MPEG |
| T10849 | 17. Jacareacanga; FLONA do Crepori; Rio das Tropas; Cotovelo; Brazil | -6.5189 | -57.4444 | MPEG |
| A23542 | 18. Rio Xingu; Jericoá, Barra do Vento ca. 65 km SE Altamira; Brazil | -3.4927 | -51.6982 | INPA |
| 562276 | 19. Altamira; 52 km SSW; E Bank Rio Xingu; Brazil | -3.6500 | -52.3700 | USNM |
| T1666 | 20. Rio Xingu; Altamira; Ilha da Taboca (UHE Belo Monte); Brazil | -3.2975 | -52.1883 | MPEG |
| A23515 | 21. Rio Iriri; Rio Xingu left margin, fluvial island; Brazil | -3.8175 | -52.6351 | INPA |
| T16723 | 22. PARNA da Serra do Pardo; Sede do ICMBio; Brazil | -5.7972 | -52.7200 | MPEG |
| T21987 | 23. Uruará; Fazenda Alcatéia; Brazil | -3.6969 | -53.5621 | MPEG |
| A23595 | 24. Rio Xingu; Vitória do Xingu; fluvial island; Brazil | -2.9293 | -51.8873 | INPA |
| T22906 | 25. Monte Alegre; Ilha Cacoal Grande; Brazil | -2.3881 | -54.3614 | MPEG |
| T8627 | 26. Almeirim; FLOTA do Paru; Brazil | -0.9333 | -53.2333 | MPEG |
| T22958 | 27. Almeirim; Rio Amazonas; Ilha do Camaleão; Brazil | -1.5375 | -52.5236 | MPEG |
| T25494 | 28. Pium; C. Pesquisa Canguçu; Furo do Sambaíba; Brazil | -9.9788 | -50.0359 | MPEG |
| T25522 | 29. Pium; Rio Javaés; Furo do Sambaíba; Brazil | -9.9788 | -50.0359 | MPEG |
| T15379 | 30. Fazenda Três Batistella; Rio Claro; Montes Claros de Goiás; Brazil | -15.9031 | -51.4106 | MPEG |
| T15535 | 31. Fazenda Lagos; Araguaiana; Brazil | -15.4447 | -51.7322 | MPEG |
| T15638 | 32. Rio do Coco right margin; Caseara; Brazil | -9.3139 | -49.9600 | MPEG |
| A16764* | ParNa Anavilhanas; Ilha Curuçá; Rio Negro; 11 km NO Novo Airão; Amazonas; Brazil | -2.53892 | -60.93007 | INPA |

Table S1.2 Data from quality assessment of reads, assembly and final loci data set for each sample used.

| **Voucher** | **No. reads** | **Quality Assessement** | | | **Assembly** | | | **Annotation** | |
| --- | --- | --- | --- | --- | --- | --- | --- | --- | --- |
|  |  | **Final no. reads** | **Excluded reads** | | **Final no. contigs** | **Unique contigs** | | **Final no. loci** | **No. loci removed** |
| A23918 | 1577660 | 1573320 | 4340 | 0.28% | 45671 | 2094 | 4.58% | 2094 | 253 |
| T16394 | 598134 | 593667 | 4467 | 0.75% | 3784 | 1957 | 51.72% | 1957 | 344 |
| A23789 | 1347408 | 1337992 | 9416 | 0.70% | 39310 | 2078 | 5.29% | 2078 | 263 |
| T22892 | 1650921 | 1645560 | 5361 | 0.32% | 4850 | 2131 | 43.94% | 2131 | 219 |
| T22832 | 1443849 | 1439300 | 4549 | 0.32% | 4744 | 2152 | 45.36% | 2152 | 221 |
| T22748 | 747271 | 742771 | 4500 | 0.60% | 3808 | 2048 | 53.78% | 2048 | 274 |
| T16263 | 814788 | 803248 | 11540 | 1.42% | 3278 | 2260 | 68.94% | 2260 | 56 |
| 80362 | 1042642 | 1036387 | 6255 | 0.60% | 18564 | 1981 | 10.67% | 1981 | 345 |
| A16536 | 1208984 | 1201272 | 7712 | 0.64% | 4021 | 2295 | 57.08% | 2295 | 56 |
| T9529 | 517790 | 515544 | 2246 | 0.43% | 3453 | 2055 | 59.51% | 2055 | 241 |
| 80628 | 752291 | 748785 | 3506 | 0.47% | 12953 | 2018 | 15.58% | 2018 | 292 |
| T15890 | 932183 | 918612 | 13571 | 1.46% | 3395 | 2272 | 66.92% | 2272 | 39 |
| 77904 | 2204341 | 2196358 | 7983 | 0.36% | 39971 | 1996 | 4.99% | 1996 | 342 |
| T19378 | 1338557 | 1334386 | 4171 | 0.31% | 4361 | 2149 | 49.28% | 2149 | 193 |
| 78435 | 1032958 | 1027154 | 5804 | 0.56% | 15915 | 1874 | 11.78% | 1874 | 442 |
| T11511 | 609753 | 607774 | 1979 | 0.32% | 3400 | 2111 | 62.09% | 2111 | 202 |
| T10849 | 261079 | 259514 | 1565 | 0.60% | 2729 | 2033 | 74.50% | 2033 | 128 |
| A23542 | 1095075 | 1091913 | 3162 | 0.29% | 17281 | 2065 | 11.95% | 2065 | 279 |
| 562276 | 1763060 | 1757218 | 5842 | 0.33% | 5352 | 2137 | 39.93% | 2137 | 210 |
| T1666 | 796034 | 793512 | 2522 | 0.32% | 3605 | 2148 | 59.58% | 2148 | 188 |
| A23515 | 1378800 | 1375278 | 3522 | 0.26% | 23517 | 2066 | 8.79% | 2066 | 284 |
| T16723 | 735686 | 731043 | 4643 | 0.63% | 3710 | 2070 | 55.80% | 2070 | 254 |
| T21987 | 2146815 | 2140234 | 6581 | 0.31% | 5658 | 2158 | 38.14% | 2158 | 198 |
| A23595 | 1028109 | 1024552 | 3557 | 0.35% | 29412 | 2044 | 6.95% | 2044 | 310 |
| T22906 | 1726513 | 1719457 | 7056 | 0.41% | 4771 | 2115 | 44.33% | 2115 | 230 |
| T8627 | 1123903 | 1120207 | 3696 | 0.33% | 4076 | 2143 | 52.58% | 2143 | 210 |
| T22958 | 1419324 | 1414962 | 4362 | 0.31% | 4591 | 2176 | 47.40% | 2176 | 179 |
| T25494 | 1089465 | 1085402 | 4063 | 0.37% | 20423 | 2059 | 10.08% | 2059 | 290 |
| T25522 | 1284224 | 1272652 | 11572 | 0.90% | 19267 | 2068 | 10.73% | 2068 | 284 |
| T15379 | 354939 | 352994 | 1945 | 0.55% | 2977 | 2079 | 69.84% | 2079 | 159 |
| T15535 | 452747 | 448873 | 3874 | 0.86% | 3386 | 2067 | 61.05% | 2067 | 207 |
| T15638 | 1280220 | 1275687 | 4533 | 0.35% | 4347 | 2119 | 48.75% | 2119 | 232 |
| A16764 | 910184 | 896821 | 13363 | 1.47% | 3474 | 2283 | 65.72% | 2283 | 50 |

Table S2. Putative Z-linked loci.

| \| **Locus** \| **Prob. identity** \| **Alignment length** \| **Mismatches** \| **Gap opens** \| **Query start** \| **Query end** \| **Subject start** \| **Subject end** \| **E-value** \| **Bit score** \| \| --- \| --- \| --- \| --- \| --- \| --- \| --- \| --- \| --- \| --- \| --- \| \| uce1212 \| 100.00 \| 28 \| 0 \| 0 \| 1 \| 28 \| 16167036 \| 16167009 \| 5.00E-06 \| 52.8 \| \| uce2548 \| 100.00 \| 30 \| 0 \| 0 \| 504 \| 533 \| 38996151 \| 38996122 \| 4.00E-07 \| 56.5 \| \| uce4312 \| 100.00 \| 31 \| 0 \| 0 \| 751 \| 781 \| 522015 \| 521985 \| 1.00E-07 \| 58.4 \| \| uce6585 \| 99.31 \| 436 \| 2 \| 1 \| 1 \| 435 \| 9732755 \| 9732320 \| 0.0 \| 787 \| \| uce106 \| 98.89 \| 539 \| 5 \| 1 \| 1 \| 538 \| 59233328 \| 59232790 \| 0.0 \| 961 \| \| uce1759 \| 98.66 \| 598 \| 3 \| 4 \| 1 \| 595 \| 177799 \| 177204 \| 0.0 \| 1055 \| \| uce4932 \| 98.44 \| 257 \| 2 \| 1 \| 443 \| 699 \| 360907 \| 360653 \| 5.00E-126 \| 451 \| \| uce998 \| 98.17 \| 709 \| 13 \| 0 \| 4 \| 712 \| 72494833 \| 72495541 \| 0.0 \| 1238 \| \| uce4932 \| 98.07 \| 569 \| 8 \| 2 \| 131 \| 699 \| 355735 \| 355170 \| 0.0 \| 987 \| \| uce802 \| 97.83 \| 736 \| 15 \| 1 \| 1 \| 736 \| 45815528 \| 45816262 \| 0.0 \| 1269 \| \| uce1555 \| 97.81 \| 729 \| 14 \| 2 \| 60 \| 787 \| 56497715 \| 56498442 \| 0.0 \| 1256 \| \| uce6478 \| 97.48 \| 516 \| 11 \| 2 \| 1 \| 515 \| 10498695 \| 10498181 \| 0.0 \| 880 \| \| uce7726 \| 97.28 \| 551 \| 14 \| 1 \| 1 \| 550 \| 59453905 \| 59453355 \| 0.0 \| 933 \| \| uce2735 \| 97.20 \| 536 \| 15 \| 0 \| 52 \| 587 \| 59768172 \| 59767637 \| 0.0 \| 907 \| \| uce3998 \| 97.14 \| 35 \| 1 \| 0 \| 6 \| 40 \| 37656184 \| 37656218 \| 4.00E-08 \| 60.2 \| \| uce2393 \| 97.11 \| 484 \| 9 \| 5 \| 202 \| 680 \| 56731639 \| 56732122 \| 0.0 \| 811 \| \| uce478 \| 97.06 \| 748 \| 19 \| 1 \| 4 \| 751 \| 59784287 \| 59783543 \| 0.0 \| 1256 \| \| uce6726 \| 96.91 \| 583 \| 18 \| 0 \| 3 \| 585 \| 53789579 \| 53788997 \| 0.0 \| 977 \| \| uce889 \| 96.74 \| 767 \| 18 \| 2 \| 2 \| 768 \| 59021993 \| 59021234 \| 0.0 \| 1271 \| \| uce642 \| 96.72 \| 580 \| 17 \| 1 \| 1 \| 578 \| 56478072 \| 56478651 \| 0.0 \| 965 \| \| uce866 \| 96.70 \| 546 \| 17 \| 1 \| 1 \| 546 \| 59428477 \| 59427933 \| 0.0 \| 907 \| \| uce1639 \| 96.56 \| 727 \| 24 \| 1 \| 15 \| 741 \| 12476932 \| 12476207 \| 0.0 \| 1203 \| \| uce864 \| 96.42 \| 755 \| 24 \| 1 \| 1 \| 755 \| 45964876 \| 45965627 \| 0.0 \| 1242 \| \| uce3026 \| 96.37 \| 524 \| 19 \| 0 \| 8 \| 531 \| 37744080 \| 37744603 \| 0.0 \| 863 \| \| uce1068 \| 96.31 \| 406 \| 15 \| 0 \| 8 \| 413 \| 53150029 \| 53149624 \| 0.0 \| 667 \| \| uce2051 \| 96.24 \| 532 \| 19 \| 1 \| 3 \| 533 \| 22280905 \| 22281436 \| 0.0 \| 870 \| \| uce7037 \| 96.24 \| 745 \| 27 \| 1 \| 1 \| 744 \| 64596622 \| 64595878 \| 0.0 \| 1219 \| \| uce7560 \| 96.22 \| 476 \| 17 \| 1 \| 1 \| 476 \| 64574127 \| 64573653 \| 0.0 \| 778 \| \| uce4080 \| 96.17 \| 418 \| 13 \| 3 \| 20 \| 435 \| 36683507 \| 36683923 \| 0.0 \| 680 \| \| uce6716 \| 96.15 \| 572 \| 16 \| 3 \| 1 \| 566 \| 62851239 \| 62850668 \| 0.0 \| 929 \| \| uce7819 \| 96.13 \| 594 \| 15 \| 3 \| 1 \| 593 \| 68066483 \| 68067069 \| 0.0 \| 963 \| \| uce1535 \| 96.06 \| 634 \| 17 \| 1 \| 178 \| 803 \| 55025925 \| 55025292 \| 0.0 \| 1026 \| \| uce5730 \| 95.86 \| 556 \| 17 \| 1 \| 1 \| 556 \| 45587517 \| 45588066 \| 0.0 \| 894 \| \| uce4844 \| 95.82 \| 789 \| 30 \| 2 \| 1 \| 787 \| 54137605 \| 54136818 \| 0.0 \| 1271 \| \| uce3958 \| 95.79 \| 594 \| 21 \| 3 \| 1 \| 593 \| 56523405 \| 56523995 \| 0.0 \| 955 \| \| uce4711 \| 95.78 \| 593 \| 21 \| 4 \| 1 \| 591 \| 54592686 \| 54592096 \| 0.0 \| 953 \| \| uce4259 \| 95.73 \| 539 \| 20 \| 2 \| 258 \| 793 \| 59380861 \| 59380323 \| 0.0 \| 865 \| \| uce5464 \| 95.69 \| 743 \| 17 \| 9 \| 16 \| 755 \| 54260687 \| 54259957 \| 0.0 \| 1181 \| \| uce5510 \| 95.63 \| 526 \| 23 \| 0 \| 31 \| 556 \| 72670355 \| 72670880 \| 0.0 \| 845 \| \| uce1178 \| 95.60 \| 637 \| 27 \| 1 \| 1 \| 637 \| 56394957 \| 56395592 \| 0.0 \| 1020 \| \| uce3318 \| 95.60 \| 636 \| 17 \| 9 \| 32 \| 656 \| 72508920 \| 72509555 \| 0.0 \| 1009 \| \| uce7384 \| 95.58 \| 746 \| 28 \| 4 \| 1 \| 742 \| 23107095 \| 23106351 \| 0.0 \| 1190 \| \| uce1915 \| 95.43 \| 635 \| 27 \| 2 \| 21 \| 654 \| 65360561 \| 65359928 \| 0.0 \| 1011 \| \| uce1036 \| 95.39 \| 499 \| 15 \| 5 \| 64 \| 557 \| 45568510 \| 45569005 \| 0.0 \| 787 \| \| uce4751 \| 95.30 \| 659 \| 30 \| 1 \| 1 \| 659 \| 34975263 \| 34975920 \| 0.0 \| 1044 \| \| uce4486 \| 95.26 \| 760 \| 32 \| 3 \| 15 \| 770 \| 59361518 \| 59360759 \| 0.0 \| 1201 \| \| uce2563 \| 95.20 \| 333 \| 15 \| 1 \| 1 \| 332 \| 53885145 \| 53884813 \| 1.00E-148 \| 525 \| \| uce5980 \| 95.04 \| 544 \| 25 \| 1 \| 1 \| 544 \| 56728540 \| 56729081 \| 0.0 \| 854 \| \| uce3046 \| 94.93 \| 691 \| 25 \| 5 \| 3 \| 688 \| 56442784 \| 56443469 \| 0.0 \| 1074 \| \| uce6725 \| 94.87 \| 643 \| 33 \| 0 \| 7 \| 649 \| 13292110 \| 13291468 \| 0.0 \| 1005 \| \| uce1630 \| 94.82 \| 599 \| 31 \| 0 \| 8 \| 606 \| 14461342 \| 14460744 \| 0.0 \| 935 \| \| uce527 \| 94.73 \| 702 \| 27 \| 9 \| 4 \| 700 \| 59718104 \| 59717408 \| 0.0 \| 1083 \| \| uce7972 \| 94.58 \| 683 \| 30 \| 3 \| 2 \| 683 \| 59331346 \| 59330670 \| 0.0 \| 1050 \| \| uce1064 \| 94.53 \| 677 \| 29 \| 5 \| 1 \| 673 \| 46671265 \| 46671937 \| 0.0 \| 1038 \| \| uce7621 \| 94.42 \| 466 \| 22 \| 4 \| 1 \| 465 \| 23082937 \| 23082475 \| 0.0 \| 713 \| \| uce309 \| 94.39 \| 748 \| 34 \| 4 \| 25 \| 765 \| 54567983 \| 54567237 \| 0.0 \| 1142 \| \| uce5334 \| 94.38 \| 587 \| 28 \| 2 \| 1 \| 582 \| 56466900 \| 56467486 \| 0.0 \| 896 \| \| uce4904 \| 94.18 \| 601 \| 34 \| 1 \| 1 \| 601 \| 60386370 \| 60385771 \| 0.0 \| 915 \| \| uce1305 \| 94.14 \| 597 \| 30 \| 4 \| 49 \| 641 \| 54092820 \| 54092225 \| 0.0 \| 904 \| \| uce6017 \| 94.10 \| 661 \| 21 \| 7 \| 4 \| 664 \| 59498809 \| 59498167 \| 0.0 \| 989 \| \| uce7164 \| 94.10 \| 559 \| 24 \| 4 \| 1 \| 558 \| 59322858 \| 59322308 \| 0.0 \| 841 \| \| uce7490 \| 94.04 \| 503 \| 24 \| 3 \| 44 \| 546 \| 59408674 \| 59408178 \| 0.0 \| 758 \| \| uce2468 \| 94.02 \| 602 \| 31 \| 4 \| 1 \| 598 \| 4985831 \| 4986431 \| 0.0 \| 907 \| \| uce6381 \| 93.85 \| 846 \| 39 \| 7 \| 1 \| 834 \| 14580865 \| 14580021 \| 0.0 \| 1262 \| \| uce2780 \| 93.77 \| 626 \| 29 \| 5 \| 1 \| 624 \| 44631804 \| 44632421 \| 0.0 \| 931 \| \| uce5514 \| 93.77 \| 642 \| 40 \| 0 \| 3 \| 644 \| 44496126 \| 44496767 \| 0.0 \| 965 \| \| uce652 \| 93.72 \| 478 \| 17 \| 7 \| 16 \| 493 \| 23260772 \| 23260308 \| 0.0 \| 704 \| \| uce3667 \| 93.67 \| 679 \| 21 \| 9 \| 1 \| 659 \| 317140 \| 316464 \| 0.0 \| 996 \| \| uce791 \| 93.66 \| 710 \| 38 \| 6 \| 7 \| 715 \| 54575135 \| 54574432 \| 0.0 \| 1055 \| \| uce6842 \| 93.60 \| 656 \| 27 \| 9 \| 17 \| 660 \| 36354997 \| 36355649 \| 0.0 \| 965 \| \| uce4022 \| 93.58 \| 685 \| 33 \| 6 \| 1 \| 677 \| 62707325 \| 62706644 \| 0.0 \| 1011 \| \| uce3493 \| 93.48 \| 721 \| 24 \| 8 \| 1 \| 699 \| 65343588 \| 65342869 \| 0.0 \| 1050 \| \| uce4770 \| 93.44 \| 671 \| 20 \| 10 \| 1 \| 669 \| 72530968 \| 72531616 \| 0.0 \| 974 \| \| uce5 \| 93.42 \| 699 \| 41 \| 4 \| 45 \| 739 \| 56538378 \| 56539075 \| 0.0 \| 1031 \| \| uce735 \| 93.32 \| 614 \| 39 \| 2 \| 1 \| 614 \| 17645218 \| 17644607 \| 0.0 \| 905 \| \| uce6910 \| 93.08 \| 650 \| 21 \| 16 \| 28 \| 676 \| 59252348 \| 59251722 \| 0.0 \| 929 \| \| uce962 \| 93.01 \| 772 \| 45 \| 7 \| 1 \| 771 \| 59048953 \| 59048190 \| 0.0 \| 1118 \| \| uce6973 \| 93.00 \| 600 \| 30 \| 8 \| 1 \| 596 \| 67851093 \| 67851684 \| 0.0 \| 865 \| \| uce7609 \| 92.96 \| 710 \| 25 \| 6 \| 1 \| 699 \| 67858567 \| 67859262 \| 0.0 \| 1011 \| \| uce3792 \| 92.95 \| 780 \| 47 \| 7 \| 2 \| 777 \| 59834364 \| 59833589 \| 0.0 \| 1129 \| \| uce838 \| 92.94 \| 524 \| 37 \| 0 \| 6 \| 529 \| 59584169 \| 59583646 \| 0.0 \| 763 \| \| uce912 \| 92.88 \| 730 \| 39 \| 4 \| 38 \| 754 \| 53908972 \| 53908243 \| 0.0 \| 1048 \| \| uce6400 \| 92.77 \| 664 \| 38 \| 3 \| 1 \| 663 \| 59733867 \| 59733213 \| 0.0 \| 952 \| \| uce4299 \| 92.68 \| 41 \| 2 \| 1 \| 61 \| 100 \| 41302337 \| 41302377 \| 1.00E-07 \| 58.4 \| \| uce6574 \| 92.60 \| 784 \| 53 \| 4 \| 1 \| 782 \| 54001363 \| 54000583 \| 0.0 \| 1122 \| \| uce1363 \| 92.59 \| 796 \| 46 \| 6 \| 13 \| 799 \| 56579337 \| 56580128 \| 0.0 \| 1131 \| \| uce2190 \| 92.57 \| 754 \| 40 \| 8 \| 1 \| 739 \| 56495533 \| 56496285 \| 0.0 \| 1068 \| \| uce5899 \| 92.54 \| 134 \| 9 \| 1 \| 1 \| 133 \| 23223742 \| 23223609 \| 1.00E-47 \| 191 \| \| uce4516 \| 92.46 \| 729 \| 43 \| 8 \| 1 \| 728 \| 6606608 \| 6605891 \| 0.0 \| 1031 \| \| uce4184 \| 92.38 \| 499 \| 34 \| 2 \| 180 \| 677 \| 62831992 \| 62831497 \| 0.0 \| 708 \| \| uce4324 \| 92.32 \| 716 \| 50 \| 5 \| 1 \| 715 \| 56469548 \| 56470259 \| 0.0 \| 1013 \| \| uce885 \| 92.30 \| 688 \| 41 \| 3 \| 24 \| 710 \| 15819633 \| 15820309 \| 0.0 \| 966 \| \| uce931 \| 92.26 \| 620 \| 40 \| 7 \| 1 \| 619 \| 54540382 \| 54539770 \| 0.0 \| 872 \| \| uce5297 \| 92.25 \| 619 \| 44 \| 3 \| 21 \| 638 \| 13499693 \| 13500308 \| 0.0 \| 874 \| \| uce995 \| 92.15 \| 548 \| 29 \| 3 \| 145 \| 679 \| 23391636 \| 23392182 \| 0.0 \| 761 \| \| uce5361 \| 92.07 \| 757 \| 49 \| 8 \| 2 \| 750 \| 56512204 \| 56512957 \| 0.0 \| 1055 \| \| uce2511 \| 92.06 \| 630 \| 40 \| 8 \| 4 \| 630 \| 9926933 \| 9926311 \| 0.0 \| 878 \| \| uce7843 \| 92.05 \| 541 \| 29 \| 6 \| 58 \| 588 \| 54121217 \| 54120681 \| 0.0 \| 749 \| \| uce221 \| 92.04 \| 729 \| 55 \| 2 \| 1 \| 726 \| 56560691 \| 56561419 \| 0.0 \| 1022 \| \| uce1492 \| 91.93 \| 694 \| 51 \| 5 \| 1 \| 690 \| 14801247 \| 14800555 \| 0.0 \| 966 \| \| uce7981 \| 91.86 \| 553 \| 35 \| 7 \| 12 \| 562 \| 62937218 \| 62936674 \| 0.0 \| 763 \| \| uce4662 \| 91.72 \| 628 \| 46 \| 5 \| 1 \| 625 \| 14000988 \| 14000364 \| 0.0 \| 867 \| \| uce736 \| 91.72 \| 870 \| 38 \| 13 \| 2 \| 871 \| 13697320 \| 13696485 \| 0.0 \| 1177 \| \| uce6757 \| 91.64 \| 550 \| 22 \| 15 \| 1 \| 548 \| 49827776 \| 49828303 \| 0.0 \| 739 \| \| uce2353 \| 91.42 \| 699 \| 52 \| 2 \| 1 \| 696 \| 59349421 \| 59348728 \| 0.0 \| 952 \| \| uce2994 \| 91.31 \| 610 \| 43 \| 8 \| 1 \| 605 \| 37503135 \| 37503739 \| 0.0 \| 824 \| \| uce7889 \| 91.26 \| 755 \| 46 \| 9 \| 4 \| 755 \| 22844774 \| 22845511 \| 0.0 \| 1011 \| \| uce4279 \| 91.23 \| 57 \| 4 \| 1 \| 1 \| 57 \| 128550 \| 128605 \| 4.00E-13 \| 76.8 \| \| uce850 \| 91.23 \| 810 \| 62 \| 5 \| 23 \| 827 \| 56713878 \| 56714683 \| 0.0 \| 1094 \| \| uce2335 \| 91.21 \| 671 \| 44 \| 6 \| 12 \| 669 \| 13335360 \| 13334692 \| 0.0 \| 898 \| \| uce3407 \| 91.05 \| 514 \| 34 \| 4 \| 4 \| 515 \| 55116083 \| 55115580 \| 0.0 \| 684 \| \| uce1378 \| 91.04 \| 603 \| 50 \| 3 \| 14 \| 613 \| 22389988 \| 22390589 \| 0.0 \| 811 \| \| uce4479 \| 91.04 \| 413 \| 34 \| 3 \| 49 \| 460 \| 54305496 \| 54305086 \| 2.00E-157 \| 555 \| \| uce6804 \| 90.89 \| 768 \| 50 \| 9 \| 1 \| 760 \| 63430677 \| 63431432 \| 0.0 \| 1013 \| \| uce1561 \| 90.56 \| 678 \| 50 \| 14 \| 138 \| 809 \| 17290788 \| 17290119 \| 0.0 \| 885 \| \| uce5218 \| 90.53 \| 739 \| 67 \| 1 \| 4 \| 739 \| 54231156 \| 54230418 \| 0.0 \| 974 \| \| uce2592 \| 90.46 \| 681 \| 51 \| 11 \| 3 \| 678 \| 23143858 \| 23143187 \| 0.0 \| 885 \| \| uce5799 \| 90.28 \| 504 \| 40 \| 7 \| 1 \| 496 \| 14646066 \| 14645564 \| 0.0 \| 651 \| \| uce645 \| 90.26 \| 390 \| 33 \| 4 \| 1 \| 385 \| 13828676 \| 13828287 \| 2.00E-142 \| 505 \| \| uce3368 \| 90.17 \| 641 \| 52 \| 5 \| 1 \| 639 \| 14820043 \| 14819412 \| 0.0 \| 824 \| \| uce5998 \| 90.09 \| 686 \| 57 \| 9 \| 1 \| 682 \| 51947278 \| 51947956 \| 0.0 \| 880 \| \| uce4445 \| 89.78 \| 626 \| 48 \| 9 \| 1 \| 623 \| 36930449 \| 36931061 \| 0.0 \| 787 \| \| uce6163 \| 89.74 \| 546 \| 48 \| 8 \| 1 \| 543 \| 12100968 \| 12100428 \| 0.0 \| 691 \| \| ALDe8 \| 89.68 \| 310 \| 30 \| 2 \| 1 \| 308 \| 11154739 \| 11154430 \| 4.00E-109 \| 394 \| \| ALDe3 \| 89.61 \| 308 \| 29 \| 3 \| 1 \| 307 \| 11156861 \| 11156556 \| 2.00E-107 \| 388 \| \| uce3917 \| 89.51 \| 696 \| 44 \| 15 \| 1 \| 668 \| 60345670 \| 60344976 \| 0.0 \| 854 \| \| uce7487 \| 89.47 \| 760 \| 57 \| 9 \| 4 \| 755 \| 53058445 \| 53057701 \| 0.0 \| 939 \| \| uce2812 \| 89.37 \| 743 \| 58 \| 9 \| 9 \| 749 \| 56559813 \| 56560536 \| 0.0 \| 915 \| \| uce5761 \| 89.21 \| 547 \| 50 \| 8 \| 1 \| 543 \| 15943109 \| 15943650 \| 0.0 \| 675 \| \| uce4261 \| 89.17 \| 628 \| 44 \| 14 \| 4 \| 618 \| 28358049 \| 28358665 \| 0.0 \| 761 \| \| uce4929 \| 89.14 \| 534 \| 33 \| 11 \| 31 \| 544 \| 71890436 \| 71890964 \| 0.0 \| 641 \| \| uce4944 \| 89.01 \| 746 \| 67 \| 10 \| 1 \| 734 \| 58964990 \| 58964248 \| 0.0 \| 909 \| \| uce6523 \| 88.89 \| 405 \| 29 \| 10 \| 162 \| 553 \| 13222685 \| 13222284 \| 4.00E-136 \| 484 \| \| ACO1e9 \| 88.79 \| 232 \| 25 \| 1 \| 22 \| 253 \| 31879634 \| 31879864 \| 7.00E-76 \| 283 \| \| uce1680 \| 88.79 \| 812 \| 61 \| 16 \| 1 \| 810 \| 14288345 \| 14287562 \| 0.0 \| 968 \| \| uce5671 \| 88.61 \| 711 \| 69 \| 5 \| 4 \| 708 \| 63219062 \| 63218358 \| 0.0 \| 854 \| \| uce1035 \| 88.60 \| 684 \| 43 \| 20 \| 1 \| 651 \| 53547277 \| 53546596 \| 0.0 \| 798 \| \| uce4878 \| 88.56 \| 507 \| 39 \| 8 \| 4 \| 491 \| 15975096 \| 15974590 \| 4.00E-170 \| 597 \| \| uce3539 \| 88.37 \| 765 \| 68 \| 14 \| 1 \| 754 \| 13792103 \| 13791349 \| 0.0 \| 900 \| \| uce1787 \| 88.27 \| 520 \| 50 \| 9 \| 3 \| 516 \| 13991183 \| 13990669 \| 2.00E-174 \| 612 \| \| uce2340 \| 88.06 \| 720 \| 75 \| 5 \| 3 \| 711 \| 22504375 \| 22505094 \| 0.0 \| 843 \| \| uce3479 \| 88.04 \| 577 \| 59 \| 9 \| 33 \| 604 \| 53587178 \| 53586607 \| 0.0 \| 675 \| \| uce6487 \| 87.78 \| 704 \| 62 \| 15 \| 1 \| 694 \| 13786782 \| 13786093 \| 0.0 \| 802 \| \| uce4200 \| 87.64 \| 518 \| 54 \| 9 \| 158 \| 672 \| 13788387 \| 13787877 \| 8.00E-169 \| 593 \| \| uce5751 \| 87.47 \| 399 \| 43 \| 6 \| 129 \| 525 \| 62082713 \| 62082320 \| 1.00E-126 \| 453 \| \| uce6527 \| 87.45 \| 534 \| 67 \| 0 \| 1 \| 534 \| 16021786 \| 16021253 \| 1.00E-175 \| 616 \| \| uce1457 \| 87.31 \| 520 \| 35 \| 18 \| 3 \| 519 \| 13593268 \| 13592777 \| 2.00E-160 \| 566 \| \| uce678 \| 86.96 \| 721 \| 78 \| 12 \| 1 \| 713 \| 13959886 \| 13959174 \| 0.0 \| 797 \| \| ACO1e10 \| 86.93 \| 306 \| 37 \| 3 \| 14 \| 317 \| 31880492 \| 31880796 \| 7.00E-93 \| 340 \| \| MUSKe5 \| 86.81 \| 326 \| 43 \| 0 \| 22 \| 347 \| 26327498 \| 26327823 \| 4.00E-100 \| 364 \| \| uce6048 \| 86.73 \| 731 \| 76 \| 10 \| 1 \| 726 \| 21853074 \| 21853788 \| 0.0 \| 793 \| \| uce7797 \| 86.59 \| 738 \| 81 \| 14 \| 1 \| 732 \| 64878365 \| 64877640 \| 0.0 \| 798 \| \| uce3277 \| 86.56 \| 707 \| 61 \| 17 \| 9 \| 684 \| 14181481 \| 14180778 \| 0.0 \| 749 \| \| uce4189 \| 86.43 \| 722 \| 78 \| 8 \| 1 \| 709 \| 15971108 \| 15970394 \| 0.0 \| 773 \| \| uce2943 \| 86.28 \| 729 \| 77 \| 14 \| 1 \| 725 \| 60458035 \| 60457326 \| 0.0 \| 771 \| \| uce7960 \| 86.20 \| 819 \| 82 \| 15 \| 2 \| 791 \| 68132663 \| 68133479 \| 0.0 \| 857 \| \| uce2486 \| 85.75 \| 737 \| 68 \| 29 \| 9 \| 718 \| 50773971 \| 50774697 \| 0.0 \| 745 \| \| uce3030 \| 85.58 \| 624 \| 62 \| 17 \| 12 \| 611 \| 45408534 \| 45409153 \| 2.00E-179 \| 628 \| \| uce5922 \| 85.19 \| 736 \| 97 \| 10 \| 1 \| 728 \| 12094258 \| 12093527 \| 0.0 \| 745 \| \| uce3417 \| 84.76 \| 656 \| 76 \| 13 \| 1 \| 640 \| 59757657 \| 59757010 \| 0.0 \| 636 \| \| uce366 \| 84.72 \| 746 \| 88 \| 18 \| 15 \| 747 \| 53924690 \| 53923958 \| 0.0 \| 723 \| \| uce6585 \| 84.38 \| 288 \| 35 \| 7 \| 425 \| 702 \| 9732297 \| 9732010 \| 1.00E-72 \| 274 \| \| uce5347 \| 84.25 \| 597 \| 84 \| 8 \| 15 \| 610 \| 53840126 \| 53839539 \| 9.00E-163 \| 573 \| \| uce2917 \| 84.21 \| 817 \| 99 \| 18 \| 1 \| 799 \| 19138770 \| 19137966 \| 0.0 \| 767 \| \| MUSKe4 \| 83.75 \| 554 \| 75 \| 12 \| 5 \| 551 \| 26326804 \| 26327349 \| 7.00E-144 \| 510 \| \| uce6032 \| 83.68 \| 582 \| 69 \| 13 \| 71 \| 649 \| 13722229 \| 13721671 \| 3.00E-148 \| 525 \| \| uce4447 \| 83.36 \| 571 \| 86 \| 6 \| 41 \| 604 \| 15584890 \| 15584322 \| 1.00E-146 \| 520 \| \| uce3181 \| 83.10 \| 639 \| 77 \| 17 \| 15 \| 636 \| 46067002 \| 46067626 \| 1.00E-156 \| 553 \| \| uce636 \| 83.10 \| 580 \| 79 \| 14 \| 1 \| 577 \| 13869543 \| 13868980 \| 7.00E-144 \| 510 \| \| uce991 \| 83.05 \| 820 \| 99 \| 24 \| 1 \| 805 \| 13737738 \| 13736944 \| 0.0 \| 708 \| \| ALDe6 \| 82.02 \| 228 \| 26 \| 7 \| 46 \| 259 \| 11155598 \| 11155372 \| 1.00E-44 \| 180 \| \| ALDe4 \| 81.78 \| 428 \| 76 \| 2 \| 1 \| 427 \| 11156291 \| 11155865 \| 7.00E-98 \| 357 \| |
| --- | --- | --- | --- | --- | --- | --- | --- | --- | --- | --- | --- | --- | --- | --- | --- | --- | --- | --- | --- | --- | --- | --- | --- | --- | --- | --- | --- | --- | --- | --- | --- | --- | --- | --- | --- | --- | --- | --- | --- | --- | --- | --- | --- | --- | --- | --- | --- | --- | --- | --- | --- | --- | --- | --- | --- | --- | --- | --- | --- | --- | --- | --- | --- | --- | --- | --- | --- | --- | --- | --- | --- | --- | --- | --- | --- | --- | --- | --- | --- | --- | --- | --- | --- | --- | --- | --- | --- | --- | --- | --- | --- | --- | --- | --- | --- | --- | --- | --- | --- | --- | --- | --- | --- | --- | --- | --- | --- | --- | --- | --- | --- | --- | --- | --- | --- | --- | --- | --- | --- | --- | --- | --- | --- | --- | --- | --- | --- | --- | --- | --- | --- | --- | --- | --- | --- | --- | --- | --- | --- | --- | --- | --- | --- | --- | --- | --- | --- | --- | --- | --- | --- | --- | --- | --- | --- | --- | --- | --- | --- | --- | --- | --- | --- | --- | --- | --- | --- | --- | --- | --- | --- | --- | --- | --- | --- | --- | --- | --- | --- | --- | --- | --- | --- | --- | --- | --- | --- | --- | --- | --- | --- | --- | --- | --- | --- | --- | --- | --- | --- | --- | --- | --- | --- | --- | --- | --- | --- | --- | --- | --- | --- | --- | --- | --- | --- | --- | --- | --- | --- | --- | --- | --- | --- | --- | --- | --- | --- | --- | --- | --- | --- | --- | --- | --- | --- | --- | --- | --- | --- | --- | --- | --- | --- | --- | --- | --- | --- | --- | --- | --- | --- | --- | --- | --- | --- | --- | --- | --- | --- | --- | --- | --- | --- | --- | --- | --- | --- | --- | --- | --- | --- | --- | --- | --- | --- | --- | --- | --- | --- | --- | --- | --- | --- | --- | --- | --- | --- | --- | --- | --- | --- | --- | --- | --- | --- | --- | --- | --- | --- | --- | --- | --- | --- | --- | --- | --- | --- | --- | --- | --- | --- | --- | --- | --- | --- | --- | --- | --- | --- | --- | --- | --- | --- | --- | --- | --- | --- | --- | --- | --- | --- | --- | --- | --- | --- | --- | --- | --- | --- | --- | --- | --- | --- | --- | --- | --- | --- | --- | --- | --- | --- | --- | --- | --- | --- | --- | --- | --- | --- | --- | --- | --- | --- | --- | --- | --- | --- | --- | --- | --- | --- | --- | --- | --- | --- | --- | --- | --- | --- | --- | --- | --- | --- | --- | --- | --- | --- | --- | --- | --- | --- | --- | --- | --- | --- | --- | --- | --- | --- | --- | --- | --- | --- | --- | --- | --- | --- | --- | --- | --- | --- | --- | --- | --- | --- | --- | --- | --- | --- | --- | --- | --- | --- | --- | --- | --- | --- | --- | --- | --- | --- | --- | --- | --- | --- | --- | --- | --- | --- | --- | --- | --- | --- | --- | --- | --- | --- | --- | --- | --- | --- | --- | --- | --- | --- | --- | --- | --- | --- | --- | --- | --- | --- | --- | --- | --- | --- | --- | --- | --- | --- | --- | --- | --- | --- | --- | --- | --- | --- | --- | --- | --- | --- | --- | --- | --- | --- | --- | --- | --- | --- | --- | --- | --- | --- | --- | --- | --- | --- | --- | --- | --- | --- | --- | --- | --- | --- | --- | --- | --- | --- | --- | --- | --- | --- | --- | --- | --- | --- | --- | --- | --- | --- | --- | --- | --- | --- | --- | --- | --- | --- | --- | --- | --- | --- | --- | --- | --- | --- | --- | --- | --- | --- | --- | --- | --- | --- | --- | --- | --- | --- | --- | --- | --- | --- | --- | --- | --- | --- | --- | --- | --- | --- | --- | --- | --- | --- | --- | --- | --- | --- | --- | --- | --- | --- | --- | --- | --- | --- | --- | --- | --- | --- | --- | --- | --- | --- | --- | --- | --- | --- | --- | --- | --- | --- | --- | --- | --- | --- | --- | --- | --- | --- | --- | --- | --- | --- | --- | --- | --- | --- | --- | --- | --- | --- | --- | --- | --- | --- | --- | --- | --- | --- | --- | --- | --- | --- | --- | --- | --- | --- | --- | --- | --- | --- | --- | --- | --- | --- | --- | --- | --- | --- | --- | --- | --- | --- | --- | --- | --- | --- | --- | --- | --- | --- | --- | --- | --- | --- | --- | --- | --- | --- | --- | --- | --- | --- | --- | --- | --- | --- | --- | --- | --- | --- | --- | --- | --- | --- | --- | --- | --- | --- | --- | --- | --- | --- | --- | --- | --- | --- | --- | --- | --- | --- | --- | --- | --- | --- | --- | --- | --- | --- | --- | --- | --- | --- | --- | --- | --- | --- | --- | --- | --- | --- | --- | --- | --- | --- | --- | --- | --- | --- | --- | --- | --- | --- | --- | --- | --- | --- | --- | --- | --- | --- | --- | --- | --- | --- | --- | --- | --- | --- | --- | --- | --- | --- | --- | --- | --- | --- | --- | --- | --- | --- | --- | --- | --- | --- | --- | --- | --- | --- | --- | --- | --- | --- | --- | --- | --- | --- | --- | --- | --- | --- | --- | --- | --- | --- | --- | --- | --- | --- | --- | --- | --- | --- | --- | --- | --- | --- | --- | --- | --- | --- | --- | --- | --- | --- | --- | --- | --- | --- | --- | --- | --- | --- | --- | --- | --- | --- | --- | --- | --- | --- | --- | --- | --- | --- | --- | --- | --- | --- | --- | --- | --- | --- | --- | --- | --- | --- | --- | --- | --- | --- | --- | --- | --- | --- | --- | --- | --- | --- | --- | --- | --- | --- | --- | --- | --- | --- | --- | --- | --- | --- | --- | --- | --- | --- | --- | --- | --- | --- | --- | --- | --- | --- | --- | --- | --- | --- | --- | --- | --- | --- | --- | --- | --- | --- | --- | --- | --- | --- | --- | --- | --- | --- | --- | --- | --- | --- | --- | --- | --- | --- | --- | --- | --- | --- | --- | --- | --- | --- | --- | --- | --- | --- | --- | --- | --- | --- | --- | --- | --- | --- | --- | --- | --- | --- | --- | --- | --- | --- | --- | --- | --- | --- | --- | --- | --- | --- | --- | --- | --- | --- | --- | --- | --- | --- | --- | --- | --- | --- | --- | --- | --- | --- | --- | --- | --- | --- | --- | --- | --- | --- | --- | --- | --- | --- | --- | --- | --- | --- | --- | --- | --- | --- | --- | --- | --- | --- | --- | --- | --- | --- | --- | --- | --- | --- | --- | --- | --- | --- | --- | --- | --- | --- | --- | --- | --- | --- | --- | --- | --- | --- | --- | --- | --- | --- | --- | --- | --- | --- | --- | --- | --- | --- | --- | --- | --- | --- | --- | --- | --- | --- | --- | --- | --- | --- | --- | --- | --- | --- | --- | --- | --- | --- | --- | --- | --- | --- | --- | --- | --- | --- | --- | --- | --- | --- | --- | --- | --- | --- | --- | --- | --- | --- | --- | --- | --- | --- | --- | --- | --- | --- | --- | --- | --- | --- | --- | --- | --- | --- | --- | --- | --- | --- | --- | --- | --- | --- | --- | --- | --- | --- | --- | --- | --- | --- | --- | --- | --- | --- | --- | --- | --- | --- | --- | --- | --- | --- | --- | --- | --- | --- | --- | --- | --- | --- | --- | --- | --- | --- | --- | --- | --- | --- | --- | --- | --- | --- | --- | --- | --- | --- | --- | --- | --- | --- | --- | --- | --- | --- | --- | --- | --- | --- | --- | --- | --- | --- | --- | --- | --- | --- | --- | --- | --- | --- | --- | --- | --- | --- | --- | --- | --- | --- | --- | --- | --- | --- | --- | --- | --- | --- | --- | --- | --- | --- | --- | --- | --- | --- | --- | --- | --- | --- | --- | --- | --- | --- | --- | --- | --- | --- | --- | --- | --- | --- | --- | --- | --- | --- | --- | --- | --- | --- | --- | --- | --- | --- | --- | --- | --- | --- | --- | --- | --- | --- | --- | --- | --- | --- | --- | --- | --- | --- | --- | --- | --- | --- | --- | --- | --- | --- | --- | --- | --- | --- | --- | --- | --- | --- | --- | --- | --- | --- | --- | --- | --- | --- | --- | --- | --- | --- | --- | --- | --- | --- | --- | --- | --- | --- | --- | --- | --- | --- | --- | --- | --- | --- | --- | --- | --- | --- | --- | --- | --- | --- | --- | --- | --- | --- | --- | --- | --- | --- | --- | --- | --- | --- | --- | --- | --- | --- | --- | --- | --- | --- | --- | --- | --- | --- | --- | --- | --- | --- | --- | --- | --- | --- | --- | --- | --- | --- | --- | --- | --- | --- | --- | --- | --- | --- | --- | --- | --- | --- | --- | --- | --- | --- | --- | --- | --- | --- | --- | --- | --- | --- | --- | --- | --- | --- | --- | --- | --- | --- | --- | --- | --- | --- | --- | --- | --- | --- | --- | --- | --- | --- | --- | --- | --- | --- | --- | --- | --- | --- | --- | --- | --- | --- | --- | --- | --- | --- | --- | --- | --- | --- | --- | --- | --- | --- | --- | --- | --- | --- | --- | --- | --- | --- | --- | --- | --- | --- | --- | --- | --- | --- | --- | --- | --- | --- | --- | --- | --- | --- | --- | --- | --- | --- | --- | --- | --- | --- | --- | --- | --- | --- | --- | --- | --- | --- | --- | --- | --- | --- | --- | --- | --- | --- | --- | --- | --- | --- | --- | --- | --- | --- | --- | --- | --- | --- | --- | --- | --- | --- | --- | --- | --- | --- | --- | --- | --- | --- | --- | --- | --- | --- | --- | --- | --- | --- | --- | --- | --- | --- | --- | --- | --- | --- | --- | --- | --- | --- | --- | --- | --- | --- | --- | --- | --- | --- | --- | --- | --- | --- | --- | --- | --- | --- | --- | --- | --- | --- | --- | --- | --- | --- | --- | --- | --- | --- | --- | --- | --- | --- | --- | --- | --- | --- | --- | --- | --- | --- | --- | --- | --- | --- | --- | --- | --- | --- | --- | --- | --- | --- | --- | --- | --- | --- | --- | --- | --- | --- | --- | --- | --- | --- | --- | --- | --- | --- | --- | --- | --- | --- | --- | --- | --- | --- | --- | --- | --- | --- | --- | --- | --- | --- | --- | --- | --- | --- | --- | --- | --- | --- | --- | --- | --- | --- | --- | --- | --- | --- | --- | --- | --- | --- | --- | --- | --- | --- | --- | --- | --- | --- | --- | --- | --- | --- | --- | --- | --- | --- | --- | --- | --- | --- | --- | --- | --- | --- | --- | --- | --- | --- | --- | --- | --- | --- | --- | --- | --- | --- | --- | --- | --- | --- | --- | --- | --- | --- | --- | --- | --- | --- | --- | --- | --- | --- | --- | --- | --- | --- | --- | --- | --- | --- | --- | --- | --- | --- | --- | --- | --- | --- | --- | --- | --- | --- | --- | --- | --- | --- | --- | --- | --- | --- | --- | --- | --- | --- | --- | --- | --- | --- | --- | --- | --- | --- | --- | --- | --- | --- | --- | --- | --- | --- | --- | --- | --- | --- | --- | --- | --- | --- | --- | --- | --- | --- | --- | --- | --- | --- | --- | --- | --- | --- | --- | --- | --- | --- | --- | --- | --- | --- | --- | --- | --- | --- | --- | --- | --- | --- | --- | --- | --- | --- | --- | --- | --- | --- | --- | --- | --- | --- | --- | --- | --- | --- | --- | --- | --- | --- | --- | --- | --- | --- | --- | --- | --- | --- | --- | --- | --- | --- | --- | --- | --- | --- | --- | --- | --- | --- | --- | --- | --- | --- | --- | --- | --- | --- | --- | --- | --- | --- | --- | --- | --- | --- | --- | --- | --- | --- | --- | --- | --- | --- | --- | --- | --- | --- | --- | --- | --- | --- | --- | --- | --- | --- | --- | --- | --- | --- | --- | --- | --- | --- | --- | --- | --- | --- | --- | --- | --- | --- | --- | --- | --- | --- | --- | --- | --- | --- | --- | --- | --- | --- | --- | --- | --- | --- | --- | --- | --- | --- | --- | --- | --- | --- | --- | --- | --- | --- | --- | --- | --- | --- | --- | --- | --- | --- | --- | --- | --- | --- | --- | --- | --- | --- | --- | --- | --- | --- | --- | --- | --- | --- | --- | --- | --- | --- | --- | --- | --- | --- | --- | --- | --- | --- | --- | --- | --- | --- | --- | --- | --- | --- | --- | --- | --- | --- | --- | --- | --- | --- | --- | --- | --- | --- | --- | --- | --- | --- | --- | --- | --- | --- | --- | --- | --- | --- | --- | --- | --- | --- | --- | --- | --- | --- | --- | --- | --- | --- | --- | --- | --- | --- | --- | --- | --- | --- |

Table S3. Summary from Bayesian clustering analyses. Mean likelihood and respective standard deviation for each number of clusters modelled performed for 10 repetitions each. The highest likelihood value is highlighted in bold letters.

| K | Mean LnP(K) | Stdev LnP(K) |
| --- | --- | --- |
| 1 | -20434.1 | 1.6352 |
| 2 | -18999.2 | 158.0722 |
| 3 | -17821.7 | 7.1187 |
| **4** | **-17520.2** | **8.6191** |
| 5 | -19079 | 3644.876 |
| 6 | -19047.2 | 2410.923 |

Table S4. Origins of expansion inferred from models considering one or multiple events. Only data sets presenting statistically significant deviations from isolation by distance equilibrium are presented (p<0.00001). Strength of the founder effect (q), founder distance (d1), r‐squared of the regression of psi *vs*. difference in distance from origin (rsq), population within the flooded forests in *Cerrado* (CE), western Amazonia (W), southeastern Amazonia (SE). * Northern Amazon River population (N; n=2) and highly admixed individual (T22906 from locality 25, not assigned to any population) had to be excluded from this analysis.

|  | **Dataset (population)** | **Latitude** | **Longitude** | **q** | **d1**  **(km)** | **rsq** |
| --- | --- | --- | --- | --- | --- | --- |
| One origin | Full dataset | -15.11522 | -49.96 | 2.0x10^-4^ | 25.31 | 0.29 |
|  | Full dataset, excluding CE | -0.9333 | -55.15765 | 1.7x10^-4^ | 30.29 | 0.15 |
| Multiple origins * | W / CE / SE  (SE) | -5.276979 | -51.6982 | 3.5x10^-4^ | 14.34 | 0.40 |

**References**

Bird, J. P., Martin, R., Akçakaya, H. R., Gilroy, J., Burfield, I. J., Garnett, S. T., … Butchart, S. H. M. (2020). Generation lengths of the world’s birds and their implications for extinction risk. *Conservation Biology*. https://doi.org/10.1111/cobi.13486

BirdLife International. (2016). Sakesphorus luctuosus*. The IUCN Red List of Threatened Species*.

Foll, M., & Gaggiotti, O. E. (2008). A genome scan method to identify selected loci appropriate for both dominant and codominant markers: a Bayesian perspective. *Genetics*, *180*, 977–993. https://doi.org/10.1534/genetics.108.092221

Lopes, L. E., & Gonzaga, L. P. (2012). Clinal pattern of morphological variation in *Sakesphorus luctuosus* (Lichtenstein, 1823), with comments on the enigmatic *Sakesphorus hagmanni* Miranda-Ribeiro, 1927 (Passeriformes: Thamnophilidae). *Zootaxa*, *3569*, 41–54. https://doi.org/10.11646/zootaxa.3569.1.3

Miguez‐Macho, G., & Fan, Y. (2012). The role of groundwater in the Amazon water cycle: 1. Influence on seasonal streamflow, flooding and wetlands. *Journal of Geophysical Research: Atmospheres*, *117*(D15). https://doi.org/10.1029/2012JD017539

Winker, K., Glenn T. C., & Faircloth B. C. (2018). Ultraconserved elements (UCEs) illuminate the population genomics of a recent, high-latitude avian speciation event. *PeerJ*, *6*:e5735. https://doi.org/10.7717/peerj.5735
